# SIRT1 Downregulation by Advanced Glycation End Products Activates RANKL‐Dependent Osteoclast Signaling and Drives Chondrocyte Senescence During Osteoarthritis Development

**DOI:** 10.1111/acel.70515

**Published:** 2026-05-01

**Authors:** Yizhou Li, Jian Wu, Rui Liu, Qiang Li, Fei Xue

**Affiliations:** ^1^ Department of Orthopedics The First Affiliated Hospital of Inner Mongolia Medical University Hohhot Inner Mongolia Autonomous Region China; ^2^ General Internal Medicine (Rheumatology and Immunology Specialty) The Second Affiliated Hospital of Inner Mongolia Medical University Hohhot Inner Mongolia Autonomous Region China; ^3^ Area A of Trauma and Orthopedics Department (Geriatric Orthopedics Department) The Second Affiliated Hospital of Inner Mongolia Medical University Hohhot Inner Mongolia Autonomous Region China

**Keywords:** advanced glycation end products, osteoclast, receptor activator of nuclear factor‐κB ligand/receptor activator of nuclear factor‐κB, sirtuin 1

## Abstract

Advanced glycation end products (AGEs) accumulate with aging and metabolic stress and are increasingly implicated in osteoarthritis (OA) pathology. However, how AGEs regulate osteoclast–chondrocyte signaling remains poorly defined. Here, we integrated proteomic and transcriptomic analyses with machine learning to identify molecular networks altered by AGEs in osteoclasts. SIRT1 emerged as a central regulator suppressed following AGE exposure. Loss of SIRT1 deacetylase activity activated the RANKL/RANK signaling pathway and enhanced osteoclast differentiation. Pharmacological inhibition of RAGE or shRNA‐mediated gene silencing restored SIRT1 expression, confirming the upstream role of AGE–RAGE signaling. In a co‐culture system, AGE‐treated osteoclasts accelerated chondrocyte senescence, as evidenced by elevated senescence markers and SASP factors. Findings were validated in vivo, where AGEs aggravated cartilage degeneration, subchondral bone alterations, and chondrocyte senescence in an OA mouse model. Collectively, these results identify an AGE‐driven SIRT1/RANKL axis that links osteoclast activation with chondrocyte aging, highlighting a critical pathway contributing to joint deterioration. Targeting this mechanism may offer new therapeutic opportunities for delaying age‐related OA progression.

## Graphical abstract

Mechanistic study of AGEs‐induced OA progression via SIRT1/RANKL/RANK signaling‐mediated osteoclast–chondrocyte communication and cellular senescence.

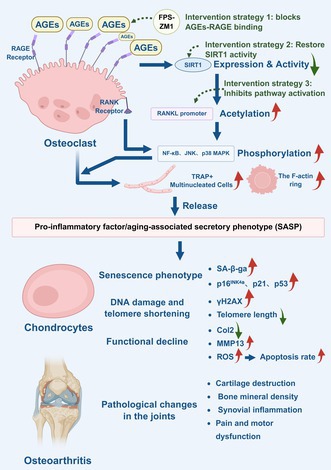

**Abbreviations**
AAV: adeno‐associated virus
AGC: automatic gain control
AGE: advanced glycation end product
BMD: bone mineral density
BV: bone volume
BV/TV: bone volume fraction
CCK‐8: cell counting kit‐8
cDNA: complementary DNA
ChIP: chromatin immunoprecipitation
Col2: type II collagen
DEG: differentially expressed gene
DEP: differentially expressed protein
FA: formic acid
FBS: fetal bovine serum
FISH: fluorescence in situ hybridization
GO: Gene Ontology
GSEA: Gene Set Enrichment Analysis
H&E: hematoxylin and eosin
HPLC: high‐performance liquid chromatography
IF: immunofluorescence
IHC: immunohistochemical
KEGG: Kyoto Encyclopedia of Genes and Genomes
LASSO: least absolute shrinkage and selection operator
M‐CSF: macrophage colony‐stimulating factor
Micro‐CT: micro‐computed tomography
MOI: multiplicity of infection
MS/MS: tandem mass spectrometer
OA: osteoarthritis
OD: optical density
PBS: phosphate‐buffered saline
PFA: paraformaldehyde
PI: propidium iodide
PPI: protein–protein interaction
RAGE: receptor for advanced glycation end products
RANKL: receptor activator of nuclear factor‐κB ligand
RIN: RNA: integrity number
RNA‐seq: RNA sequencing
ROS: reactive oxygen species
SASP: senescence‐associated secretory phenotype
SA‐β‐gal: SA‐β‐galactosidase
shRNA: short hairpin RNA
SIRT1: sirtuin 1
SPF: specific pathogen‐free
Tb.N: trabecular number
Tb.Sp: trabecular separation
TIFs: telomere dysfunction‐induced foci
TRAP: tartrate‐resistant acid phosphatase
α‐MEM: α‐minimum essential medium

## 1 Introduction

Osteoarthritis (OA) is a prevalent degenerative joint disorder marked by articular cartilage degeneration, synovial inflammation, aberrant bone remodeling, and joint dysfunction (Guo et al. 2025; Liu et al. 2025; Wei and Bai 2016). With accelerating population aging and the rising burden of metabolic disorders, OA incidence continues to increase, substantially diminishing quality of life in middle‐aged and older adults and representing a major global public health challenge (Velasquez and Katz 2010; Jeong and Lee 2021). Recent studies suggest that OA is not merely a consequence of localized cartilage wear but a “whole‐joint disease” involving interactions among multiple intra‐articular cell types (Yao et al. 2023; Kuang et al. 2024). Among these, the crosstalk between osteoclasts and chondrocytes has drawn increasing attention in the context of OA pathogenesis (Dai et al. 2024; Guo et al. 2022). Dysregulated osteoclast function not only exacerbates imbalanced bone remodeling but may also influence chondrocyte biology through secreted factors, promoting their senescence and apoptosis (Xiao et al. 2023). Therefore, elucidating the molecular mechanisms underlying osteoclast–chondrocyte interactions is crucial for understanding the pathological progression of OA.

Advanced glycation end products (AGEs) are relatively stable metabolites generated under hyperglycemia and oxidative stress, and they are closely linked to multiple chronic degenerative disorders (Reddy et al. 2022; Corica et al. 2022). Their pathogenic actions largely occur through interaction with the receptor for AGEs (RAGE), which triggers oxidative stress and pro‐inflammatory signaling, disrupting cellular homeostasis, and accelerating tissue degeneration (Zhou et al. 2024; Vitorakis and Piperi 2024). In the joint environment, elevated AGE levels are considered a significant contributor to the onset and progression of OA (He et al. 2022; Shane Anderson and Loeser 2010). Evidence also indicates that AGEs promote chondrocyte inflammation and apoptosis, while facilitating osteoclast differentiation and bone resorption (Yang et al. 2022; Ding et al. 2020). However, whether AGEs affect chondrocyte function via osteoclast‐mediated mechanisms, thereby contributing to the progression of OA, remains insufficiently studied. Clarifying the mechanisms of AGE‐mediated intercellular communication will provide insights into OA pathology from the perspective of the joint microenvironment as a whole.

Sirtuin 1 (SIRT1) is an NAD^+^‐dependent deacetylase that regulates diverse physiological functions, including cellular metabolism, antioxidant responses, and aging‐related pathways (Liao et al. 2024; Aksoy et al. 2006). Studies have shown that SIRT1 exerts protective effects in articular cartilage, and its downregulation is closely associated with the progression of OA (Leah 2011; Terauchi et al. 2016). The Receptor Activator of Nuclear Factor‐κB Ligand (RANKL)/RANK signaling axis is a key regulator of osteoclast differentiation and bone resorption (Cao 2018; Ikebuchi et al. 2018). Emerging evidence suggests that SIRT1 suppresses RANKL expression primarily through its histone deacetylase activity at gene promoter regions or via modulation of upstream transcription factors, thereby regulating osteoclast differentiation and function (Cai et al. 2023; Qu et al. 2018). It is hypothesized that under stimulation by AGEs, reduced SIRT1 activity may relieve its suppressive effect on RANKL, leading to activation of the RANKL/RANK pathway, enhanced osteoclast differentiation, and, through intercellular signaling, increased chondrocyte senescence and dysfunction, ultimately accelerating OA progression (Yang et al. 2022; Ding et al. 2020). However, this hypothesis has not yet been fully validated and requires multidimensional experimental support.

With the rapid advancement of multi‐omics technologies, integrative analyses combining proteomics and transcriptomics offer a comprehensive approach to deciphering the molecular response networks elicited by cellular stimulation (Sanches et al. 2024; Clark et al. 2023). Furthermore, incorporating machine learning algorithms facilitates the identification of key regulatory targets from complex datasets, thereby enhancing the precision and depth of research (Kumar et al. 2024; Li et al. 2022). On this basis, the establishment of a co‐culture system involving osteoclasts and chondrocytes enables a more physiologically relevant simulation of intra‐articular cellular interactions, allowing for the assessment of osteoclast‐induced changes in chondrocyte function. Additionally, in vivo animal model studies can validate the roles of key signaling pathways, thereby bridging the gap between experimental discovery and clinical application. Therefore, an integrated approach combining multi‐omics profiling, bioinformatics analyses, and complementary in vitro and in vivo experiments offers a robust framework for mechanistic investigation.

In summary, this study aims to investigate whether AGEs activate the RANKL/RANK signaling pathway through SIRT1‐mediated deacetylation of RANKL, thereby promoting osteoclast differentiation and inducing chondrocyte senescence via intercellular interaction, ultimately accelerating the progression of OA. We employ an integrative proteomic and transcriptomic approach to identify differentially regulated signaling pathways in osteoclasts upon AGE stimulation, followed by machine learning to screen for key molecular targets. The regulatory function of the AGEs–SIRT1–RANKL signaling axis in osteoclast–chondrocyte crosstalk is examined using RT‐qPCR, Western blot (WB), targeted molecular interventions, and co‐culture systems. Furthermore, animal models are utilized to confirm the in vivo relevance of this pathway in OA pathogenesis. This study systematically elucidates how AGE‐induced changes in osteoclast function affect chondrocyte senescence and OA progression through the SIRT1/RANKL axis, advancing the mechanistic understanding of OA and highlighting potential therapeutic targets for AGE‐related degenerative joint disorders with translational significance.

## 2 Materials and Methods

### 2.1 Ethical Statement

All animal procedures were conducted in strict accordance with applicable ethical guidelines and regulations for animal experimentation. All protocols were approved by the Institutional Animal Care and Use Committee (IACUC) (Ethical Approval Number: Y.2025130). Animals were maintained under humane conditions, with measures taken to reduce pain and distress. At the conclusion of the experiment, mice were euthanized under anesthesia.

### 2.2 Transcriptome Sequencing and Data Quality Control

Total RNA was extracted from osteoclasts in the Control (*n* = 3) and AGEs groups, as well as from chondrocytes co‐cultured with Control or AGE‐treated osteoclasts, using TRIzol reagent (Thermo Fisher Scientific, USA). RNA concentration and purity were measured with a Qubit RNA Assay Kit (Shanghai Biogene Biotech, HKR2106‐01, China) on a Qubit 2.0 Fluorometer (Life Technologies, Q33216, USA), a NanoDrop spectrophotometer (IMPLEN, USA), and an RNA Nano 6000 Assay Kit on a Bioanalyzer 2100 system (Agilent, 5067‐1511, USA), respectively. Samples meeting the following thresholds were included: concentration ≥ 20 ng/μL, OD260/280 > 2.0, RNA integrity number (RIN) ≥ 7.0, and 28S/18S ≥ 1.0.

For library preparation, 3 μg of total RNA from each sample was used to construct complementary DNA (cDNA) libraries with the NEBNext Ultra RNA Library Prep Kit for Illumina (NEB, E7435L, China). Library quality and fragment size distribution were assessed using the Agilent 2100 Bioanalyzer. Indexed libraries were amplified and clustered on the cBot system with the TruSeq PE Cluster Kit v3‐cBot‐HS (Illumina, USA), followed by paired‐end sequencing (125/150 bp) on an Illumina HiSeq 550 platform.

Raw reads were assessed with FastQC (v0.11.8). Adapter sequences and poly(A) tails were removed using Cutadapt (v1.18). Reads containing > 5% ambiguous bases (N) were discarded using Perl scripts. Quality filtering was performed with the FASTX Toolkit (v0.0.13), retaining reads in which at least 70% of bases exhibited a Phred score > 20. Clean paired‐end reads were further corrected using BBMap and subsequently mapped to the mouse reference genome with HISAT2 (v0.7.12).

### 2.3 Proteomic Sequencing and Analysis

Total protein was isolated from osteoclasts in the Control (*n* = 3) and AGEs groups, as well as from chondrocytes co‐cultured with Control or AGE‐treated osteoclasts, using RIPA buffer supplemented with protease inhibitors. Lysates were sonicated intermittently (30 s every 5 min, three cycles) to ensure complete disruption. Protein concentration was measured by BCA assay and calibrated against a standard curve. Samples were adjusted to pH 8.0 and digested with trypsin at a 1:50 enzyme‐to‐protein ratio at 37°C for 16 h. Peptides were desalted using ZipTip C18 columns and subsequently analyzed by HPLC–MS/MS. Protein identification and label‐free quantification were performed with MaxQuant software.

After desalting with 0.1% formic acid (FA), peptides were labeled using iTRAQ reagents and subjected to mass spectrometric analysis. Samples were analyzed in triplicate on a QSTAR Elite Hybrid MS (Applied Biosystems/MDS‐SCIEX) coupled to an online HPLC system (Shimadzu, Japan). For each run, 30 μL of peptide solution was loaded onto a self‐packed C18 nano‐column (75 μm × 15 cm, 5 μm; New Objectives, USA) and separated using a 90‐min gradient composed of mobile phase A (0.1% FA and 2% acetonitrile) and mobile phase B (0.1% FA and 100% acetonitrile) at a flow rate of 0.2 μL/min. Mass spectrometry was performed in positive ion mode across an m/z range of 300–2000. Precursor ions with charge states of +2 to +4 and ion counts > 5 were selected for fragmentation. Dynamic exclusion was set to 30 s with a precursor mass tolerance of 30 mDa. Information‐dependent acquisition (IDA) was applied with automatic collision energy and MS/MS accumulation (fragment intensity multiplier 20; maximum accumulation time 2 s). Three technical replicates per sample were conducted to enhance proteome coverage and data reliability. Selected proteins were further validated by WB. For DIA‐based analysis, MS parameters included: full MS scan (m/z 350–1500, resolution 120,000, AGC target 4 × 10^5^, maximum injection time 50 ms); HCD‐MS/MS (resolution 30,000, AGC target 1 × 10^5^, normalized collision energy 33); and 47 overlapping DIA windows with 1 m/z overlap. An indexed retention time (iRT) kit (Biognosys AG, Switzerland) was added for calibration. DIA data were processed using Spectronaut v13 for normalization and relative quantification.

### 2.4 Transcriptomic and Proteomic Data Analysis

Differential expression analyses for both transcriptomic and proteomic datasets were conducted in R using the limma package. Differentially expressed genes (DEGs) and proteins (DEPs) between the Control group (*n* = 3) and AGE‐treated osteoclasts were identified with the criteria |log_2_FC| > 1 and *p* < 0.01. For chondrocytes co‐cultured with Control versus AGE‐treated osteoclasts, DEGs and DEPs were screened using |log_2_FC| > 1 and *p* < 0.05. DEG volcano plots were generated with ggplot2, and Venn diagrams were created using the vennDiagram package. Gene set enrichment analysis (GSEA) was conducted using the clusterProfiler package, which was also applied for Gene Ontology (GO) and Kyoto Encyclopedia of Genes and Genomes (KEGG) pathway enrichment analyses. Protein–protein interaction (PPI) networks were generated through the GeneMANIA online platform.

### 2.5 Least Absolute Shrinkage and Selection Operator (LASSO) Regression Analysis

To identify key genes associated with disease status, LASSO regression analysis was conducted using the “glmnet” package in R. Ten‐fold cross‐validation was performed using the cv.glmnet function, with the regularization parameter alpha set to 1 to apply the L1 penalty. The family parameter was set to “binomial” to fit a logistic regression model appropriate for binary classification. A total of 100 lambda (λ) values were automatically generated by setting nlambda = 100, and the optimal lambda value was determined during cross‐validation based on the minimum deviance. The lambda value corresponding to the lowest cross‐validation error (lambda.min) was selected as the optimal penalty. Genes retaining non‐zero coefficients at the optimal λ value were defined as key candidate variables.

### 2.6 Cell Culture and Differentiation

Osteoclast precursors were obtained by isolating bone marrow–derived mononuclear cells from C57BL/6 mice (strain 213; Vital River Laboratory Animal Technology Co. Ltd., Beijing, China). Femurs and tibias were aseptically collected, rinsed with phosphate‐buffered saline (PBS), and trimmed at the epiphyses. Bone marrow was flushed from the shafts with sterile PBS to obtain marrow cells. Cells were cultured in α‐minimum essential medium (α‐MEM; Gibco, 12571063, USA) supplemented with macrophage colony‐stimulating factor (M‐CSF; PeproTech, 315‐02‐1MG, USA; 30 ng/mL), 10% fetal bovine serum (FBS; Gibco, A5256701, USA), and 1% penicillin/streptomycin (Sigma‐Aldrich, 15140148, USA). Cultures were maintained at 37°C in 5% CO_2_ for 3 days to allow adherence of mononuclear cells. Thereafter, receptor activator of nuclear factor‐κB ligand (RANKL; PeproTech, 315‐11‐10UG, USA; 50 ng/mL) was added, and cells were cultured for an additional 4 days to induce osteoclast differentiation (Wu et al. 2022).

### 2.7 Cell Treatment and Grouping

Two primary osteoclast groups were defined: Control and AGEs. Cells in the Control group were maintained under standard culture conditions without AGE stimulation. In the AGEs group, 200 μg/mL of AGEs (2221‐BSA, BioVision, USA) were added on day 4 of culture and maintained for 48 h. Each group was tested in three independent biological replicates to ensure reproducibility.

To assess the effects of exogenous AGEs and the RAGE inhibitor FPS‐ZM1 on SIRT1 expression in osteoclasts, two additional groups were defined: AGEs and AGEs‐i. The AGEs group was treated with 200 μg/mL AGEs on day 4 of culture for 48 h. The AGEs‐i group received the same concentration of AGEs, along with 10 μM/mL FPS‐ZM1 (11909, Cayman Chemical, USA), at the same time point for 48 h. All groups were maintained in α‐MEM containing 10% FBS and 1% penicillin/streptomycin.

To obtain chondrocytes, primary chondrocytes were isolated from the articular cartilage of neonatal C57BL/6 mice (within 24–48 h post‐natal). Under sterile conditions, the knee joints were exposed, and cartilage tissue was harvested specifically from the distal femoral condyles and proximal tibial plateaus. To ensure purity and avoid contamination from the growth plate or subchondral bone, the dissection was performed under a stereomicroscope. Only the translucent, superficial uncalcified cartilage layers were carefully peeled off using micro‐forceps, while the opaque deeper layers and vascularized bone tissue were strictly excluded. The harvested cartilage was rinsed in cold PBS, cut into small pieces, and incubated with trypsin–EDTA at 37°C for 15 min to remove adherent soft tissues. Subsequently, the tissue was digested with collagenase (2 mg/mL; 1148090, Sigma‐Aldrich, USA) for 2 h at 37°C in Dulbecco's Modified Eagle Medium (DMEM; 11965092, Gibco, USA) containing 10% FBS, 100 U/mL penicillin, and 100 μg/mL streptomycin. Isolated cells were cultured at 37°C in a humidified atmosphere containing 5% CO_2_, and the medium was refreshed every 2–3 days. First‐passage chondrocytes reaching approximately 85% confluence were selected for subsequent experiments. A Transwell chamber system (3401, Corning, USA) was used to establish an indirect co‐culture, physically separating osteoclasts (in the upper chamber) and chondrocytes (in the lower chamber) while allowing the exchange of soluble factors. All assays were conducted in triplicate (Ji et al. 2022; Zhang et al. 2018).

### 2.8 Collection of Osteoclast Conditioned Medium (OC‐CM)

Osteoclasts were induced and differentiated as described above. On day 4, cells were treated with AGEs (200 μg/mL) or vehicle for 48 h. To exclude the interference of serum proteins, the cells were washed twice with PBS and incubated in serum‐free α‐MEM for an additional 24 h. Supernatants were harvested, centrifuged at 2000 rpm for 10 min to eliminate cellular debris, and passed through a 0.22‐μm filter. The collected OC‐CM was aliquoted and stored at −80°C until further use.

### 2.9 Antibody Neutralization Assay

Chondrocytes were plated in 6‐well plates and grown to approximately 60% confluence. The medium was then replaced with a 1:1 mixture of fresh DMEM and OC‐CM. For cytokine neutralization assays, OC‐CM was pre‐incubated at 37°C for 1 h with neutralizing antibodies against IL‐6, TNF‐α, or RANKL (each at 1 μg/mL). Species‐matched isotype IgG (1 μg/mL) served as the control. The pretreated conditioned medium was subsequently added to chondrocytes and maintained for 48 h. Cells were then harvested for SA‐β‐gal staining and WB analysis to assess senescence‐related phenotypes.

### 2.10 SIRT1 Activity Assay

Treated cells were lysed with RIPA buffer containing protease inhibitors to extract total protein. Endogenous SIRT1 protein was enriched by immunoprecipitation using anti‐SIRT1 antibody (#8469, 1:100, Cell Signaling Technology) coupled to Protein A/G magnetic beads (88,803, Thermo Scientific). Immunopurified SIRT1 was incubated with the fluorogenic substrate (acetylated p53 peptide) and NAD^+^ from the SIRT1 Activity Assay Kit (BML‐AK555‐0001, Enzo Life Sciences) at 37°C for 20 min. After terminating the reaction, fluorescence was recorded on a microplate reader (Ex 360 nm and Em 460 nm). SIRT1 activity was quantified as relative fluorescence intensity.

### 2.11 F‐Actin Ring Staining

To evaluate the degree of osteoclast differentiation, F‐actin ring formation was assessed using phalloidin staining. Cells were fixed with 4% paraformaldehyde (PFA, 47608, Sigma‐Aldrich, USA) for 20 min and washed three times with PBS (5 min each). Cells were then incubated with phalloidin (A12381, Invitrogen, USA) diluted 1:40 for 30 min at room temperature. After staining, cells were washed three more times with PBS and mounted using an anti‐fade mounting medium (H‐1900, Vectashield, USA). F‐actin ring structures were observed and captured using a fluorescence microscope (Nikon, Japan) at 200× magnification. The number and morphology of F‐actin rings were quantified and analyzed in at least five independent fields of view to ensure statistical reliability.

### 2.12 Tartrate‐Resistant Acid Phosphatase (TRAP) Staining

For cellular TRAP staining, cultures were fixed and incubated in tartrate buffer at 37°C for 30 min, followed by staining with TRAP solution (Beyotime, P0332, China) for 1 h in the dark. Images were acquired using an Axiovert 40 CFL microscope (Carl Zeiss). Multinucleated TRAP‐positive cells containing ≥ 3 nuclei were defined as mature osteoclasts. Osteoclast number and relative cell area per well were quantified using ImageJ. For tissue TRAP staining, paraffin‐embedded sections were deparaffinized, rehydrated, and treated with a TRAP staining kit (PUMOKE, PMK0467B, China). Sections were incubated in working solution at 37°C for approximately 20 min, rinsed, and counterstained with hematoxylin. After dehydration and mounting, TRAP‐positive cells were visualized under a light microscope and quantified relative to the total cell population within the specified regions.

### 2.13 Chromatin Immunoprecipitation (ChIP)‐qPCR Detection of RANKL Acetylation Levels

ChIP was performed using the EZ‐ChIP Kit (Millipore, 17‐371). Briefly, mouse cells were crosslinked with 1% formaldehyde to fix protein‐DNA interactions. After quenching the reaction, cells were lysed using SDS Lysis Buffer. Chromatin was fragmented to 200–500 bp by sonication at 4°C for 2 h (30‐s on/off cycles) using a Diagenode Bioruptor sonicator. Sheared chromatin was incubated overnight at 4°C with 2 μg of specific antibody prebound to Protein G magnetic beads (ThermoFisher, 10003D) in ChIP dilution buffer. For enrichment of acetylated histone‐associated DNA fragments, an anti‐H3K27ac antibody (Cell Signaling Technology, #9649, 1:50) was used. Immunocomplexes were sequentially washed with low‐salt, high‐salt, LiCl, and TE buffers to remove nonspecific bindings. The bound chromatin was eluted and subjected to reverse crosslinking. RNA and proteins were removed by RNase A and Proteinase K digestion, respectively. DNA was purified using the QIAquick PCR Purification Kit (QIAGEN, 28104). qPCR was performed on the purified DNA using primers specific to the promoter region of mouse RANKL. Primer sequences were as follows: Forward: GTCTTCCCAATAGCCCGTGA; Reverse: GCTCTAGTGAACTCCGTCCG.

### 2.14 RT‐qPCR Analysis of Gene Expression

Total RNA from tissues and cultured cells was isolated using TRIzol reagent (Thermo Fisher, USA). Complementary DNA (cDNA) was synthesized with the PrimeScript RT reagent kit (Takara, Japan). Quantitative real‐time PCR was performed using SYBR Premix Ex Taq II (Takara, Japan) on an ABI 7500 Real‐Time PCR System (Thermo Fisher, USA). The amplification protocol included an initial denaturation at 95°C for 30 s, followed by 40 cycles of 95°C for 5 s and 60°C for 30 s. A melting curve analysis was subsequently conducted at 95°C for 15 s, 60°C for 60 s, and 90°C for 15 s to confirm specificity. GAPDH served as the internal reference gene. Each sample was tested in triplicate, and all experiments were independently repeated three times. Gene expression levels were calculated using the 2^−ΔΔCt^ method. Primer sequences are listed in Table S1.

### 2.15 WB Analysis

Total protein was extracted from cells or tissues using RIPA lysis buffer (Thermo Fisher Scientific, USA), and concentrations were determined by BCA assay (Thermo Fisher Scientific, USA). Equal amounts of protein (30 μg per sample) were resolved by SDS–PAGE and transferred to PVDF membranes (Millipore, USA). Membranes were blocked with 5% nonfat milk (Thermo Fisher Scientific, LP0033B, USA) and incubated overnight at 4°C with primary antibodies against SIRT1, RANKL, RANK, NF‐κB, p‐NF‐κB, JNK, p‐JNK, p38 MAPK, and p‐p38 MAPK (details in Table S2). After washing, membranes were incubated with HRP‐conjugated anti‐rabbit IgG secondary antibody (Cell Signaling Technology, 7074P2, USA). Protein bands were visualized using enhanced chemiluminescence (ECL) substrate and imaged with a Bio‐Rad chemiluminescence detection system. Band intensities were quantified using ImageJ software (v1.52, NIH, USA).

### 2.16 Enzyme‐Linked Immunosorbent Assay (ELISA)

IL‐6, TNF‐α, IL‐1β, and soluble RANKL (sRANKL) levels in OC‐CM were quantified using commercially available ELISA kits. Optical density was measured at 450 nm using a microplate reader. To account for variations in cell density, cytokine levels were normalized to the total protein content of the corresponding osteoclast lysates.

### 2.17 Lentiviral Transduction

Lentiviral vectors targeting SIRT1 and RANKL, as well as a negative control (sh‐NC), were obtained from HANBIO (Shanghai, China). Cells were plated in 6‐well plates at 1 × 10^5^ cells per well and cultured for 24 h. When confluence reached ~75%, cells were transduced with lentivirus at an MOI of 10 (titer ~5 × 10^6^ TU/mL) in the presence of polybrene (5 μg/mL; Merck, TR‐1003). After 48 h, stable transductants were selected using puromycin (10 μg/mL; Sigma‐Aldrich, USA) for at least 7 days. shRNA sequences are listed in Table S3, and plasmid constructs were supplied by HANBIO (Wu et al. 2022).

Experimental Grouping: The following treatment groups were established: AGEs‐i group: osteoclasts treated with 200 μg/mL AGEs (Sigma‐Aldrich, USA) and 10 μM/mL RAGE inhibitor FPS‐ZM1 (Cayman Chemical, USA) for 48 h; AGEs‐i + sh‐SIRT1 group: same treatment as AGEs‐i, with additional SIRT1 knockdown; AGEs‐i + sh‐SIRT1 + sh‐RANKL group: same treatment as above, with combined SIRT1 and RANKL knockdown. All assays were conducted in triplicate to ensure statistical robustness.

### 2.18 Senescence Marker Detection

Chondrocyte senescence was evaluated using an SA‐β‐galactosidase (SA‐β‐gal) Staining Kit (Beyotime, C0602, China). Blue‐stained (SA‐β‐gal–positive) cells were imaged under a light microscope (Leica Microsystems, Germany), and senescence was quantified as the percentage of SA‐β‐gal–positive cells.

### 2.19 Immunofluorescence (IF) Detection of Senescence Markers and DNA Damage

IF staining was performed to assess the expression of proteins associated with cellular senescence and DNA damage in both cells and tissue samples. The following markers were evaluated: senescence markers p16^INK4a^ (ab211542, 1:100, Abcam), P21 (ab188224, 1:500, Abcam), P53 (ab26, 1:100, Abcam). SASP factors included TNF‐α (Abcam, ab183218, 1:5000), IL‐6 (Abcam, ab290735, 1:50), and IL‐1β (Abcam, ab283818, 1:50). DNA damage markers included γH2AX (Abcam, ab81299) and TRF‐1 (Abcam, ab192629). Cells were fixed with 4% PFA for 15 min, washed with PBS, and permeabilized with 0.1% Triton X‐100 for 10 min. After blocking at room temperature for 1 h, cells were incubated overnight at 4°C with primary antibodies. The next day, the samples were washed three times with PBS and incubated for 1 h in the dark at room temperature with fluorophore‐conjugated secondary antibodies: Goat Anti‐Rabbit IgG H&L (Alexa Fluor 594, ab150080) or Goat Anti‐Rabbit IgG H&L (Alexa Fluor 488, ab150077). Nuclei were counterstained with DAPI, and samples were mounted for imaging using a fluorescence microscope (Leica Microsystems, Germany). For tissue staining, samples were fixed in 4% PFA for 48 h, dehydrated, embedded in paraffin, and sagittally sectioned at a thickness of 5 μm. After deparaffinization and rehydration, antigen retrieval was performed using citrate buffer at elevated temperatures. After cooling, sections were processed using the same permeabilization, blocking, antibody incubation, and imaging procedures as described for cells.

### 2.20 Detection of γH2AX Foci and Telomere Dysfunction‐Induced Foci (TIFs)

To evaluate DNA damage and telomere dysfunction, γH2AX foci and TIFs were assessed 48 h after transfection. Chondrocytes were plated on glass coverslips and maintained for 24 h at 37°C in an atmosphere containing 5% CO_2_. Cells were subsequently transfected with target constructs or matched control vectors using lipofection reagents. After 48 h of transfection, the cells were fixed with 4% PFA in PBS for 10 min and then permeabilized with 0.25% Triton X‐100 in PBS for an additional 10 min. Following PBS washes, cells were blocked in PBS containing 1% BSA and 0.1% Tween‐20 for 1 h. Cells were then incubated with a mouse monoclonal antibody against TRF‐1 (TRF‐78, ab10579, Abcam; 1:1000) and a rabbit polyclonal antibody against phosphorylated H2AX (γH2AX, Ser139; #9718, Cell Signaling Technology; 1:100) to detect co‐localized DNA damage foci.

### 2.21 Telomere Length Measurement and Telomere Fluorescence In Situ Hybridization (FISH)

Telomere FISH was conducted using a peptide nucleic acid (PNA) probe (Pangene, F1002, China). Chondrocytes were seeded on glass coverslips in 6‐well plates and allowed to attach for 2 h at 37°C. Cells were subsequently swollen in KCl solution, fixed in methanol/acetic acid (3:1), rehydrated in PBS, refixed with 4% PFA, and dehydrated through a graded ethanol series. Coverslips were incubated in hybridization buffer containing 70% formamide, 10 mM sodium phosphate (NaHPO_4_, pH 7.4), 10 mM NaCl, and 20 mM Tris (pH 7.5). Genomic DNA was heat‐denatured at 80°C for 5 min and then incubated with the PNA telomere probe for 2 h at room temperature to allow hybridization. Following post‐hybridization washes, coverslips were mounted with DAPI‐containing Vectashield medium (Vector Laboratories, USA) and examined under a confocal microscope.

### 2.22 Cell Counting Kit‐8 (CCK‐8) Assay

To assess cell proliferation and viability, the CCK‐8 assay was performed. Cells were seeded in 96‐well plates and treated as indicated. After 24 h, fresh medium containing CCK‐8 reagent (Beyotime, C0037, China) was added to each well and incubated for 1 h. Absorbance was measured at 450 nm using a microplate reader (BioTek, USA), and OD values were used to determine relative cell viability/proliferation.

### 2.23 Flow Cytometry for Apoptosis Detection

Apoptotic cells were quantified by flow cytometry using Annexin V‐FITC/propidium iodide (PI) dual staining. Cells were harvested, washed twice with PBS, and stained with an Annexin V‐FITC/PI apoptosis detection kit (Beyotime, China). Cells were suspended in binding buffer, stained with Annexin V‐FITC (1:100) and PI (1:1000) for 15 min in the dark, and immediately subjected to flow cytometric analysis (BD FACSCalibur, BD, USA) to determine apoptotic fractions.

### 2.24 Measurement of Intracellular Reactive Oxygen Species (ROS)

Intracellular ROS production was evaluated using a commercial ROS detection kit (Beyotime, S0033S, China). After treatment, cells were incubated with 5 μM dihydroethidium for 20 min, washed twice with PBS, and imaged under a fluorescence microscope (Leica Microsystems, Germany). Relative ROS levels were quantified based on fluorescence intensity.

### 2.25 Animal Model Establishment and Experimental Grouping

Eight‐week‐old male C57BL/6 mice (Beijing Vital River Laboratory Animal Technology Co. Ltd., China) were utilized to generate a collagenase‐induced OA model. OA was established by intra‐articular injection of 10 units of collagenase type VII (C0773, Sigma‐Aldrich, USA) dissolved in 10 μL sterile phosphate‐buffered saline (PBS, pH 7.4) into the right knee joint via the parapatellar tendon approach. Mice were euthanized 5 weeks after induction.

After successful modeling, animals were randomly divided into four groups (*n* = 6 per group): Control (saline injection); (2) AGEs, receiving AGEs; (3) AGEs‐i, receiving AGEs combined with the RAGE inhibitor FPS‐ZM1; and (4) AGEs‐i + AAV‐SIRT1, receiving AGEs, FPS‐ZM1, and AAV‐SIRT1. Experimental agents were administered via intra‐articular injection. To ensure chemical stability and biological activity, all formulations were freshly prepared prior to administration or stored in aliquots to avoid repeated freeze–thaw cycles. AGEs (BSA‐AGEs) were dissolved in sterile PBS (pH 7.4) and injected at a dose of 50 μg/10 μL per mouse twice weekly. FPS‐ZM1 was initially dissolved in dimethyl sulfoxide (DMSO) to prepare a stock solution and subsequently diluted with sterile saline to the working concentration, with the final DMSO concentration maintained below 1% to minimize solvent‐related toxicity. FPS‐ZM1 was co‐administered with AGEs at 10 μg/10 μL per mouse.

For gene regulation, AAV‐SIRT1 vectors were preserved at −80°C and thawed on ice immediately before use. Viral suspensions were prepared in sterile PBS supplemented with 0.001% Pluronic F‐68 to reduce aggregation and surface adsorption. A single intra‐articular injection of AAV‐SIRT1 (1 × 10^11^ vg/mL, 10 μL) was administered during the first week of treatment (Park et al. 2020).

All mice were maintained under specific pathogen‐free (SPF) conditions with unrestricted access to food and water.

### 2.26 Pain Sensitivity Test

Mice were placed individually in transparent enclosures on a perforated metal floor and allowed to acclimate for at least 10 min before testing. Mechanical nociceptive thresholds were assessed using the von Frey filament up‐down method (NC12775, YUYAN INSTRUMENTS, China). The 50% paw withdrawal threshold was determined according to the Dixon up–down method based on the sequence of positive and negative withdrawal responses to graded mechanical stimulation.

### 2.27 Locomotor Function Test

Mice were habituated to the testing environment for 2 h prior to the open‐field assay. Spontaneous activity was assessed using the VersaMax Animal Activity Monitoring System (AccuScan Instruments, USA). Each mouse was placed in the center of a chamber (29 × 22 × 22 cm) and permitted to move freely for 60 min. The total distance traveled was recorded automatically for analysis.

### 2.28 Micro‐Computed Tomography (Micro‐CT) Scanning

Knee joints were collected and fixed in 4% PFA for 48 h. Subchondral bone architecture was assessed using a micro‐computed tomography scanner (Bruker, USA) at an isotropic resolution of 18 μm. The affected joint from each mouse was scanned to quantify bone mineral density (BMD), bone volume (BV), bone volume fraction (BV/TV), and trabecular microstructural parameters.

### 2.29 Hematoxylin and Eosin (H&E) Staining

To evaluate the cellular structure and inflammatory status of joint tissues, H&E staining was performed. Knee joint tissues were fixed in 4% PFA for 24 h and decalcified in 10% EDTA for 20 days. Specimens were subsequently dehydrated in graded ethanol, cleared, and embedded in paraffin. Serial sections (4 μm) were prepared, floated on a warm water bath, and mounted onto glass slides, followed by incubation at 60°C to ensure complete paraffin removal. Sections were stained with hematoxylin (H3136, Sigma‐Aldrich, USA) for 4 min and counterstained with eosin (E4009, Sigma‐Aldrich, USA) for 1.5 min. After dehydration and clearing, slides were mounted with neutral resin. Histopathological features were observed and imaged using a light microscope (Olympus, Japan).

### 2.30 Immunohistochemical (IHC) Staining

IHC staining was performed to detect MMP13 (ab315267, 1:100, Abcam) and type II collagen (Col2, ab34712, 1:100, Abcam). Tissue sections were incubated with primary antibodies overnight at 4°C. After washing, sections were incubated with goat anti‐rabbit IgG secondary antibody (ab6721, 1:1000, Abcam). Signals were developed using a DAB kit (P0203, Beyotime, China) and counterstained with hematoxylin. Slides were dehydrated, cleared, and mounted. Positive staining was observed and quantified under a light microscope.

### 2.31 Safranin O‐Fast Green Staining

Safranin O–Fast Green staining was performed to evaluate proteoglycan loss and cartilage degeneration. Knee specimens were fixed in 4% PFA for 24 h at 4°C, decalcified in 10% EDTA (pH 7.4) for 2 weeks, embedded in paraffin, and sagittally sectioned at 4 μm. Sections were stained with Fast Green (PH1852, PHYGENE, China) for 5 min to label collagenous structures, briefly differentiated in 1% acetic acid, and subsequently stained with Safranin O (PH1852, PHYGENE, China) for 30 min to visualize proteoglycans. Slides were then dehydrated, cleared, and mounted. Cartilage damage was graded using the OARSI scoring system (0–6) across four compartments: medial femoral condyle, medial tibial plateau, lateral femoral condyle, and lateral tibial plateau. Scores ranged from 0 (normal cartilage) to 6 (> 75% lesion extension into calcified cartilage). Two blinded observers independently evaluated each section, and the mean value was used for analysis. OA severity was defined by the highest score among the four quadrants.

### 2.32 Statistical Analysis

All data are presented as mean ± standard deviation (SD) from at least three independent experiments. For comparisons between two groups, an unpaired Student's *t*‐test was used. One‐way analysis of variance (ANOVA) was employed for comparisons among three or more groups. If ANOVA indicated significant differences, Tukey's HSD post hoc test was performed for multiple comparisons. For data that did not meet normality or homogeneity of variance assumptions, nonparametric tests (Mann–Whitney *U* or Kruskal–Wallis) were applied as appropriate. Statistical analyses were performed with GraphPad Prism 9.5.0 and R (v4.2.1). A two‐sided *p* < 0.05 was considered statistically significant.

## 3 Results

### 3.1 Multi‐Omics Analysis Reveals That AGEs Promote Osteoclast Differentiation by Inhibiting SIRT1

To examine the impact of AGEs on osteoclastogenesis, osteoclast precursors were differentiated with M‐CSF and RANKL and treated with AGEs (Figure S1A). Phenotypic evaluation demonstrated that AGEs markedly promoted cytoskeletal remodeling, characterized by enhanced F‐actin ring formation (Figure S1B), and facilitated osteoclast differentiation, as indicated by an increased number of TRAP‐positive multinucleated cells (Figure S1C).

**FIGURE 1:**
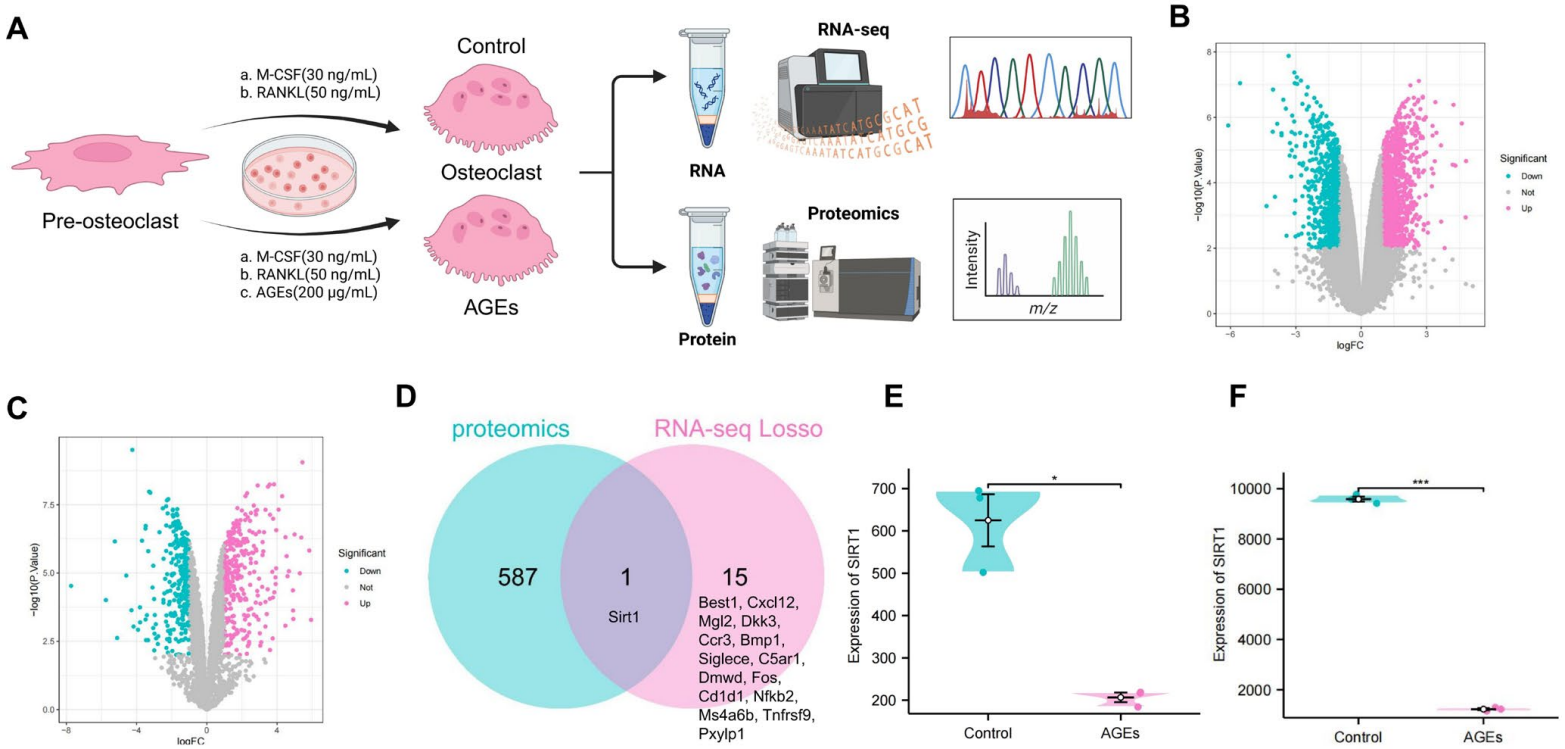
Effects of AGEs on the proteomic and transcriptomic profiles of osteoclasts and multi‐omics integration analysis. (A) Schematic workflow of proteomic and transcriptomic sequencing and multi‐omics integration analysis; (B) Number of DEGs between the Control and AGEs groups based on RNA‐seq; (C) Number of DEPs identified by proteomic sequencing between the Control and AGEs groups; (D) Venn diagram showing key genes identified by integrative analysis; (E) Transcriptomic expression level of SIRT1; (F) Proteomic expression level of SIRT1. Experiments were performed in triplicate. *Indicates significance between two groups (*p < 0.05, ***p < 0.001).

To uncover the molecular drivers of this phenotype, we performed an integrated transcriptomic and proteomic analysis (Figure 1A). RNA sequencing (RNA‐seq) identified 1701 differentially expressed genes (DEGs) (Figure 1B), while proteomic analysis revealed 588 differentially expressed proteins (DEPs) (Figure 1C). Functional enrichment analyses (GO and KEGG) indicated that these alterations were primarily associated with ECM organization, structural remodeling, and MAPK signaling pathways (Figure S1D,G). GSEA further confirmed the upregulation of ECM‐related gene sets and cell cycle pathways in the AGEs group (Figure S2).

To identify core regulatory targets from these extensive datasets, we applied a LASSO regression model to the transcriptomic data to eliminate redundant features, narrowing down to 16 candidate genes (Figure S1E,F). Intersection of these 16 feature genes with the DEPs via a Venn diagram pinpointed SIRT1 as a unique overlapping target (Figure 1D). Further validation showed that AGE exposure markedly reduced SIRT1 expression at both the mRNA (Figure 1E) and protein levels (Figure 1F). Collectively, these data suggest that AGEs promote osteoclast activation by suppressing SIRT1, thereby modulating ECM remodeling and signaling networks.

### 3.2 AGEs Downregulate SIRT1 via RAGE and Activate the RANKL/RANK Pathway

To investigate the regulatory effect of AGEs on SIRT1 expression, RT‐qPCR and WB were performed to assess SIRT1 levels in osteoclasts from the Control and AGEs groups. AGE stimulation significantly reduced SIRT1 mRNA (Figure S3A) and protein expression (Figure S3B) compared with controls. These results aligned with the transcriptomic and proteomic findings, confirming that AGEs exert a repressive effect on SIRT1 expression. In addition, SIRT1 enzymatic activity was markedly decreased in the AGEs group (Figure S3C), further supporting the inhibitory effect of AGEs on both the expression and function of SIRT1.

**FIGURE 2:**
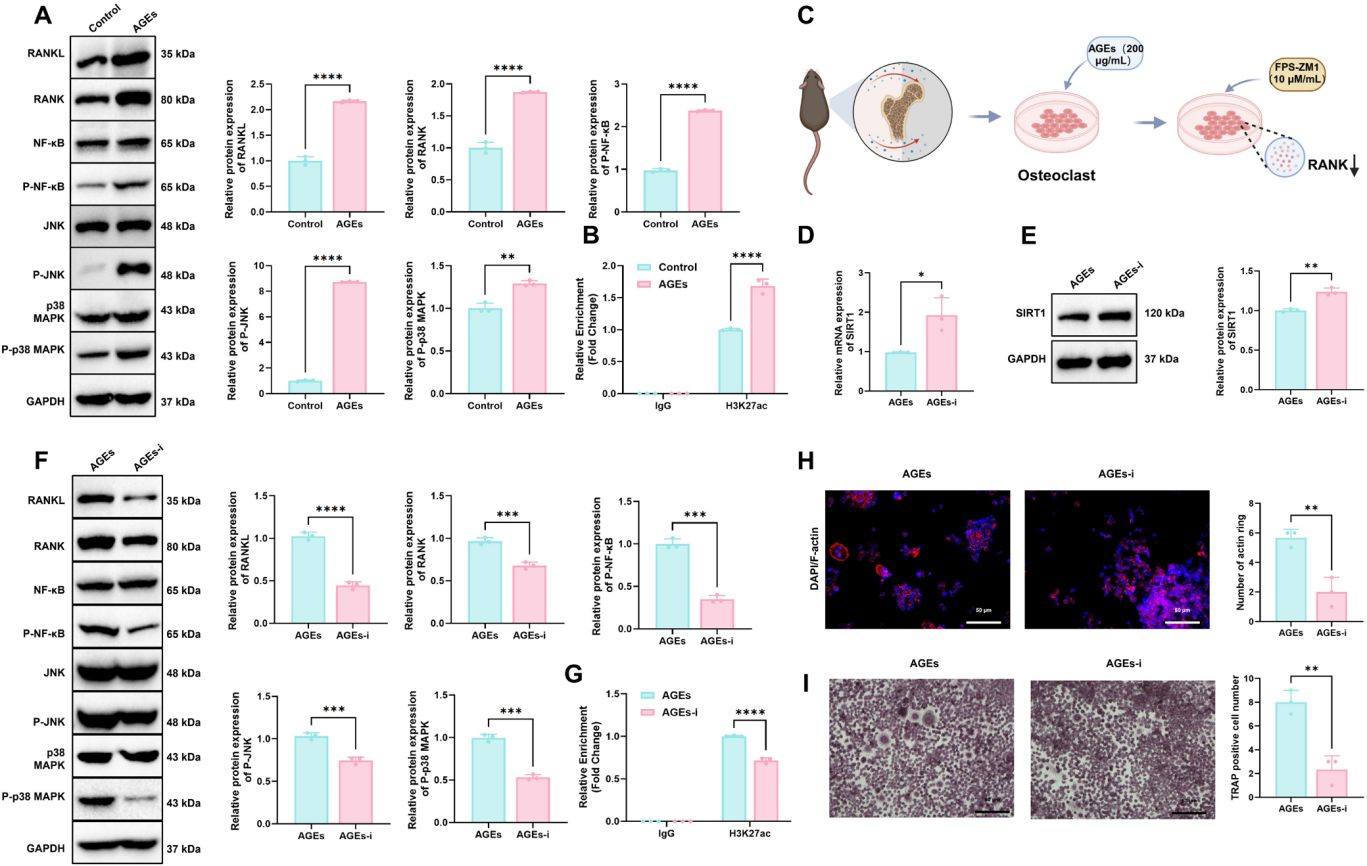
Regulatory effects of AGEs on SIRT1 expression and the RANKL/RANK pathway and the modulatory role of RAGE inhibition. (A) Western blot analysis of phosphorylated RANKL, RANK, NF‐κB, JNK, and p38 MAPK in the Control and AGEs groups; (B) Acetylation level of RANKL; (C) Schematic of experimental design involving RAGE inhibitor treatment; (D) RT‐qPCR analysis of SIRT1 mRNA expression in AGEs and AGEs‐i groups; (E) Western blot analysis of SIRT1 protein expression in AGEs and AGEs‐i groups; (F) Western blot analysis of RANKL, RANK, and downstream signaling molecules (total and phosphorylated NF‐κB, JNK, and p38 MAPK) in AGEs and AGEs‐i groups; (G) RANKL acetylation level; (H) F‐actin ring staining to assess cytoskeletal reorganization in osteoclasts (bar: 50 μm); (I) TRAP staining to evaluate osteoclast differentiation (bar: 50 μm). Experiments were performed in triplicate. *Indicates significance between two groups (*p < 0.05, **p < 0.01, ***p < 0.001, ****p < 0.0001).

Previous evidence indicates that SIRT1 inhibits osteoclast formation by deacetylating the RANKL promoter and suppressing its transcription (Cai et al. 2023). Therefore, we examined the impact of AGEs on the RANKL/RANK signaling pathway. RT‐qPCR results revealed that AGEs significantly upregulated RANKL and RANK mRNA expression compared with the Control group (Figure S3D). WB analysis confirmed elevated protein levels of RANKL, RANK, and phosphorylated downstream effectors, including NF‐κB, JNK, and p38 MAPK, in the AGEs group (Figure 2A). Furthermore, ChIP‐qPCR analysis revealed that the enrichment of acetylated histone H3 (H3K27ac) at the Tnfsf11 (RANKL) promoter region was significantly increased (Figure 2B). These findings indicate that AGEs suppress SIRT1 expression and activity, thereby reducing SIRT1‐mediated histone deacetylation at the RANKL promoter, leading to transcriptional upregulation of RANKL and activation of the RANKL/RANK signaling pathway. These findings suggest that AGEs suppress SIRT1 expression and activity, thereby releasing their inhibitory effect on the RANKL/RANK pathway and subsequently activating NF‐κB, JNK, and p38 MAPK signaling.

To verify whether exogenous AGEs regulate SIRT1 expression and osteoclast differentiation via RAGE‐mediated signaling, AGE‐induced osteoclasts were treated with the RAGE inhibitor FPS‐ZM1 (10 μM/mL) (Figure 2C). SIRT1 activity, RANKL/RANK signaling, and osteoclast differentiation were assessed by RT‐qPCR, WB, and histochemical staining. Compared with the AGEs group, SIRT1 mRNA (Figure 2D) and protein levels (Figure 2E) were markedly elevated in the AGEs‐i group, accompanied by restoration of enzymatic activity (Figure S3E). Meanwhile, RT‐qPCR revealed that mRNA levels of RANKL and RANK were significantly decreased in the AGEs‐i group (Figure S3F). WB analysis further demonstrated that phosphorylation levels of RANKL, RANK, and downstream signaling molecules NF‐κB, JNK, and p38 MAPK were all significantly reduced (Figure 2F), along with decreased RANKL acetylation (Figure 2G). These findings indicate that the RAGE inhibitor restores SIRT1 expression, thereby suppressing activation of the RANKL/RANK pathway and its downstream effectors. Additionally, F‐actin ring staining and TRAP staining were used to assess osteoclast differentiation. Compared with the AGEs group, the AGEs‐i group showed substantially diminished F‐actin ring formation (Figure 2H) along with a pronounced reduction in TRAP‐positive multinucleated osteoclasts (Figure 2I). These results suggest that inhibition of RAGE restores SIRT1 expression, suppresses the RANKL/RANK pathway, and effectively attenuates osteoclast differentiation.

Together, these findings demonstrate that AGEs may suppress SIRT1 expression and activity via RAGE‐mediated signaling, inhibiting SIRT1‐mediated histone deacetylation at the RANKL promoter, upregulate RANKL expression, and activate the RANKL/RANK pathway and its downstream molecules, thereby promoting osteoclast differentiation.

### 3.3 AGEs Suppress SIRT1 Expression to Activate the RANKL/RANK Axis and Promote Osteoclast Differentiation

**FIGURE 3:**
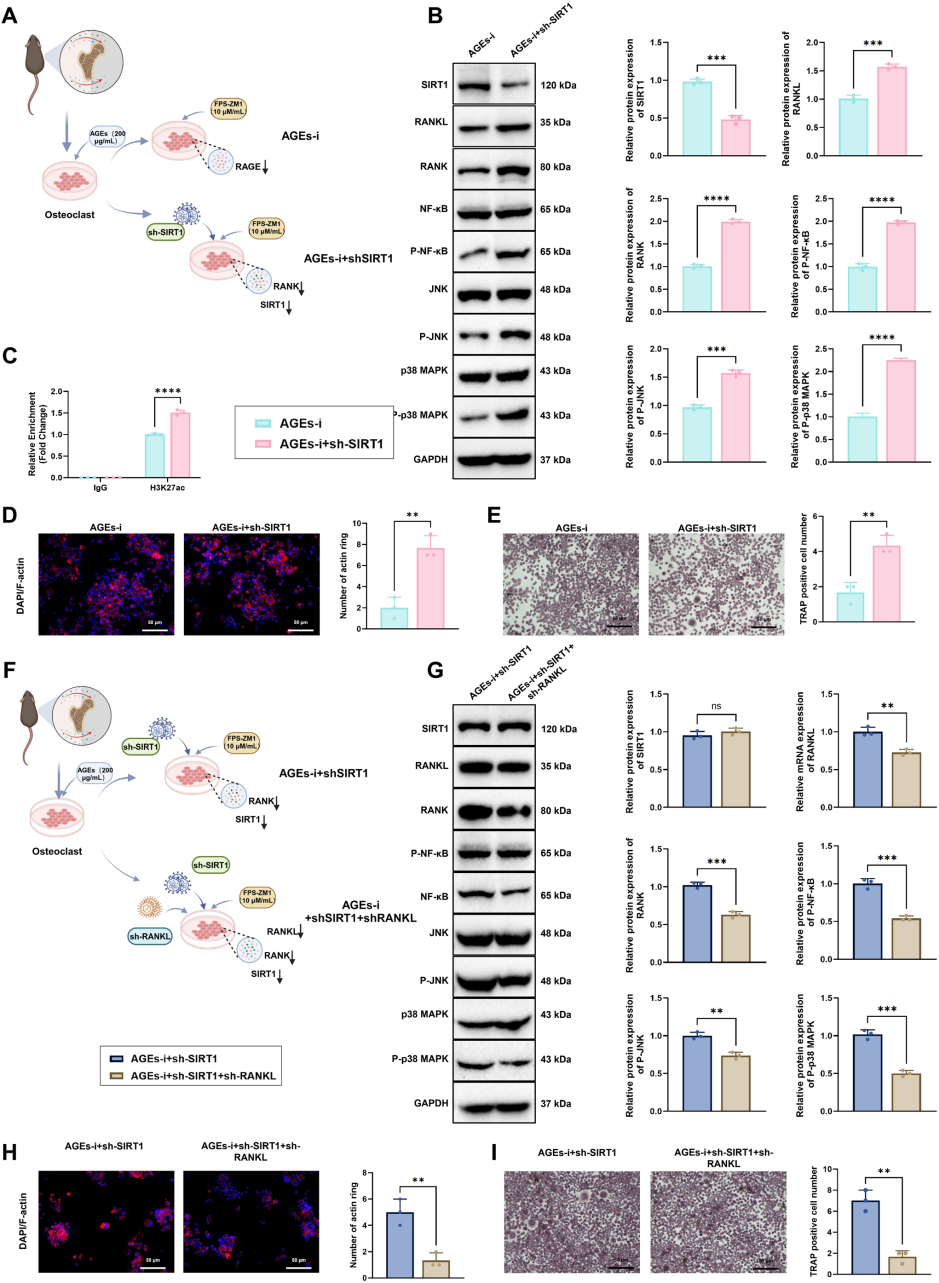
Effects of SIRT1 and RANKL silencing on AGEs‐induced osteoclast differentiation. (A) Schematic diagram of the SIRT1 silencing experimental design; (B) Western blot analysis of SIRT1, RANKL, RANK, and phosphorylated NF‐κB, JNK, and p38 MAPK protein levels in AGEs‐i and AGEs‐i+sh‐SIRT1 groups; (C) Assessment of RANKL acetylation levels; (D) F‐actin ring staining to evaluate cytoskeletal remodeling of osteoclasts in AGEs‐i and AGEs‐i+sh‐SIRT1 groups, bar = 50 μm; (E) TRAP staining to assess osteoclast differentiation in AGEs‐i and AGEs‐i+sh‐SIRT1 groups, bar = 50 μm; (F) Schematic diagram of the RANKL silencing experimental design; (G) Western blot analysis of SIRT1, RANKL, RANK, and phosphorylated NF‐κB, JNK, and p38 MAPK protein levels in AGEs‐i+sh‐SIRT1 and AGEs‐i+sh‐SIRT1+sh‐RANKL groups; (H) F‐actin ring staining to evaluate cytoskeletal remodeling in AGEs‐i+sh‐SIRT1 and AGEs‐i+sh‐SIRT1+sh‐RANKL groups, bar = 50 μm; (I) TRAP staining to assess osteoclast differentiation in AGEs‐i+sh‐SIRT1 and AGEs‐i+sh‐SIRT1+sh‐RANKL groups, bar = 50 μm. All experiments were performed in triplicate. *Indicates comparison between two groups; ns, *p* > 0.05, **p < 0.01, ***p < 0.001, ****p < 0.0001.

To further confirm that exogenous AGEs promote osteoclast differentiation via SIRT1‐mediated regulation of the RANKL/RANK signaling pathway, SIRT1‐silenced osteoclasts were constructed using lentiviral transduction. Two experimental groups were established: AGEs‐i and AGEs‐i+sh‐SIRT1 (Figure 3A). Silencing efficiency was evaluated by RT‐qPCR and WB, showing that sh‐SIRT1‐2 achieved stronger suppression than sh‐SIRT1‐1 (Figure S4A,B). Accordingly, sh‐SIRT1‐2 was used in subsequent experiments and designated as sh‐SIRT1. Compared with the AGEs‐i group, the AGEs‐i+sh‐SIRT1 group exhibited significantly decreased SIRT1 mRNA (Figure S4C) and protein expression (Figure 3B). Meanwhile, mRNA levels of RANKL and RANK were significantly increased (Figure S4C), and WB analysis confirmed elevated phosphorylation of RANKL, RANK, and downstream signaling molecules NF‐κB, JNK, and p38 MAPK (Figure 3B), along with increased RANKL acetylation (Figure 3C). These findings indicate that SIRT1 knockdown abolishes the suppressive effect of RAGE inhibition on RANKL/RANK signaling, thereby restoring pathway activation.

F‐actin ring staining and TRAP staining were further used to assess osteoclast differentiation. Relative to the AGEs‐i group, SIRT1 knockdown markedly increased F‐actin ring assembly (Figure 3D) and significantly elevated the number of TRAP‐positive multinucleated cells (Figure 3E). These results indicate that silencing SIRT1 negates the protective effect of RAGE inhibition, reactivates RANKL/RANK signaling, and promotes osteoclast differentiation.

To clarify whether the RANKL/RANK axis mediates AGEs/SIRT1‐driven osteoclastogenesis, RANKL‐silenced osteoclasts were generated by lentiviral transduction. Two groups were established: AGEs‐i + sh‐SIRT1 and AGEs‐i + sh‐SIRT1 + sh‐RANKL (Figure 3F). Knockdown efficiency analysis showed that sh‐RANKL‐2 achieved stronger suppression than sh‐RANKL‐1 (Figure S4D,E); therefore, sh‐RANKL‐2 was used in subsequent experiments and designated as sh‐RANKL. SIRT1 expression, RANKL/RANK pathway activity, and osteoclast differentiation were assessed using RT‐qPCR, WB, and cytochemical staining. Compared with the AGEs‐i + sh‐SIRT1 group, the AGEs‐i + sh‐SIRT1 + sh‐RANKL group showed no significant change in SIRT1 mRNA (Figure S4F) or protein levels (Figure 3G). However, RANKL and RANK mRNA levels were significantly reduced (Figure S4F), and WB revealed decreased expression of RANKL, RANK, and the phosphorylated forms of downstream effectors NF‐κB, JNK, and p38 MAPK (Figure 3G), indicating effective suppression of the RANKL/RANK signaling pathway by RANKL knockdown. Furthermore, F‐actin ring formation and TRAP staining were used to evaluate osteoclast differentiation. Compared to the AGEs‐i + sh‐SIRT1 group, the AGEs‐i + sh‐SIRT1 + sh‐RANKL group exhibited markedly reduced F‐actin ring formation (Figure 3H) and fewer TRAP‐positive multinucleated cells (Figure 3I), confirming that RANKL knockdown significantly inhibits osteoclast differentiation via suppression of the RANKL/RANK pathway.

Collectively, these findings further confirm that AGEs promote osteoclast differentiation by downregulating SIRT1 expression, which subsequently activates the RANKL/RANK signaling pathway and its downstream effectors.

### 3.4 AGEs Induce Chondrocyte Senescence and Dysfunction via Osteoclast‐Mediated Crosstalk

**FIGURE 4:**
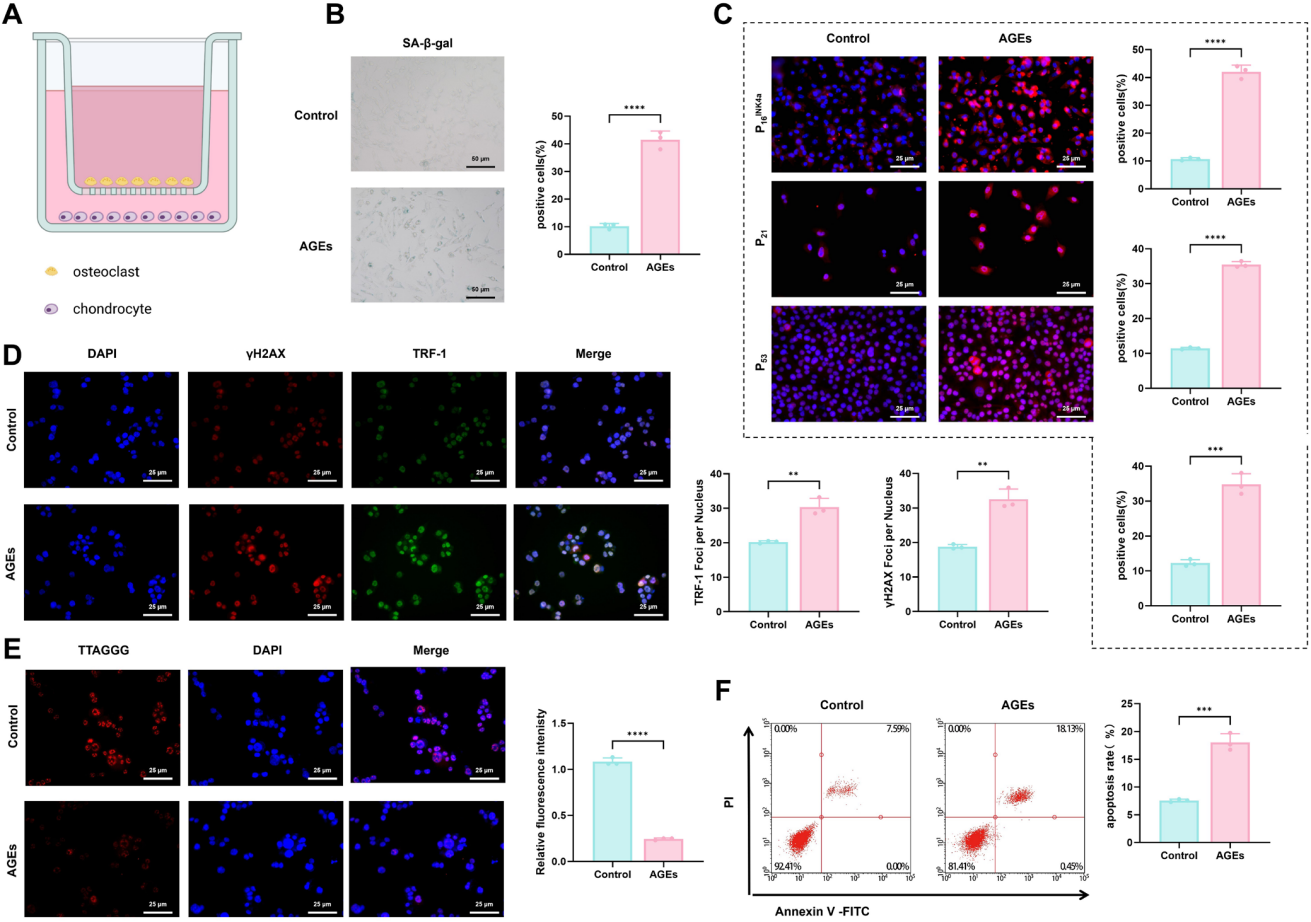
Effects of AGE‐treated osteoclasts on chondrocyte senescence. (A) Schematic diagram of the co‐culture experimental design between osteoclasts and chondrocytes; (B) SA‐β‐gal staining to assess the proportion of senescent chondrocytes in control and AGEs groups, bar = 50 μm; (C) IF analysis of chondrocyte senescence markers p16^INK4a^, p21, and p53 in control and AGEs groups, bar = 25 μm; (D) IF analysis of DNA damage marker γH2AX and telomere‐associated protein TRF‐1 in control and AGEs groups, bar = 25 μm; (E) Representative fluorescence microscopy images from telomere FISH analysis, bar = 25 μm; (F) Flow cytometric analysis of apoptosis rates in chondrocytes from control and AGEs groups. All experiments were performed in triplicate. *Indicates comparison between two groups; **p < 0.01, ***p < 0.001, ****p < 0.0001.

To investigate the effects of AGE‐treated osteoclasts on chondrocyte senescence, co‐culture experiments were conducted using Transwell chambers, with osteoclasts from the Control and AGE groups co‐cultured with chondrocytes (Figure 4A). Chondrocyte senescence phenotypes, DNA damage, oxidative stress, telomere alterations, proliferation, and apoptosis were assessed using SA‐β‐gal staining, immunofluorescence, ROS assays, telomere FISH, CCK‐8, and flow cytometry, respectively.

SA‐β‐gal staining showed a markedly increased proportion of senescent chondrocytes in the AGEs group compared with the Control group (Figure 4B). IF analysis demonstrated increased expression of senescence markers p16^INK4a^, p21, and p53 (Figure 4C), along with elevated levels of SASP factors TNF‐α, IL‐6, and IL‐1β (Figure S5A) in the AGEs group, indicating robust chondrocyte senescence induced by AGEs‐treated osteoclasts. In addition, γH2AX and the telomere‐binding protein TRF‐1 were significantly upregulated in the AGEs group (Figure 4D), accompanied by evident telomere shortening (Figure 4E). ROS analysis indicated enhanced ROS generation in chondrocytes exposed to AGE‐treated osteoclasts (Figure S5B), suggesting that oxidative stress and genomic instability contribute to the senescence phenotype. Functional analyses showed that chondrocyte proliferation was significantly reduced, as determined by CCK‐8 assay (Figure S5C), while flow cytometry detected a marked increase in apoptotic cells (Figure 4F). These findings indicate that AGE‐treated osteoclasts exacerbate chondrocyte dysfunction by promoting senescence, inducing DNA damage and oxidative stress, inhibiting proliferation, and triggering apoptosis.

To further delineate the specific paracrine mediators responsible for this intercellular crosstalk, we analyzed the secretome of AGE‐stimulated osteoclasts. ELISA analysis revealed that, in addition to soluble RANKL (sRANKL), AGEs treatment significantly upregulated the secretion of pro‐inflammatory cytokines, including IL‐6, TNF‐α, and IL‐1β (Figure S6A–D). To determine the functional contribution of these cytokines, we performed neutralization assays using specific antibodies. Pre‐incubation of AGE‐OC‐CM with neutralizing antibodies against IL‐6 or TNF‐α resulted in a partial but significant reduction in chondrocyte senescence compared to the IgG isotype control group (Figure S6E). However, the rescue effect observed with cytokine neutralization was less pronounced than the direct inhibition of the RANKL signaling axis. These data suggest that while osteoclast‐derived inflammatory cytokines synergistically contribute to the paracrine induction of senescence, the RANKL‐mediated pathway plays a dominant role in this pathological process.

Together, AGE‐treated osteoclasts significantly impair chondrocyte function through the induction of cellular senescence, genomic damage, oxidative stress, reduced proliferation, and increased apoptosis.

### 3.5 Multi‐Omics Analysis Reveals That AGEs‐Induced Osteoclast Differentiation Promotes Chondrocyte Senescence

**FIGURE 5:**
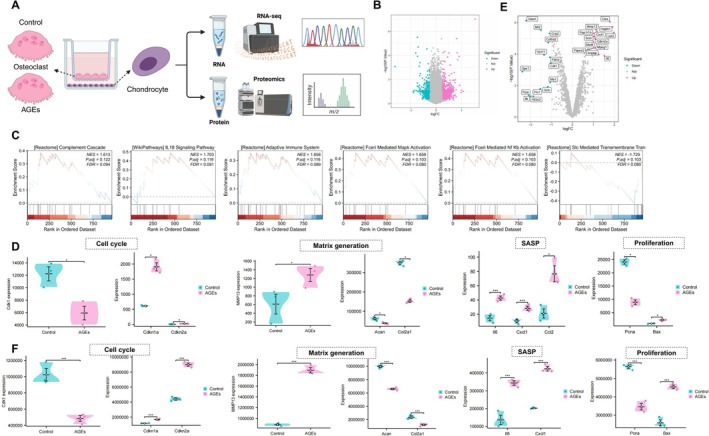
Multi‐omics analysis reveals the effects of AGE‐treated osteoclasts on chondrocyte senescence. (A) Schematic diagram of the multi‐omics analysis workflow; (B) Volcano plot of DEGs in chondrocytes from control and AGEs groups (RNA‐seq); (C) GSEA analysis of NF‐κB signaling, MAPK signaling, and cellular senescence‐related pathways based on RNA‐seq data; (D) Expression levels of genes related to cell cycle, SASP, matrix synthesis, and cell proliferation in RNA‐seq data; (E) Volcano plot of DEPs in chondrocytes from control and AGEs groups (proteomics); (F) Expression levels of proteins related to cell cycle, SASP, matrix synthesis, and cell proliferation in proteomics data. Each group, *n* = 3. * indicates comparison between two groups; *p < 0.05, **p < 0.01, ***p < 0.001.

To comprehensively investigate the effects of AGE‐treated osteoclasts on chondrocyte senescence, integrated transcriptomic and proteomic analyses were performed on co‐cultured chondrocytes (Figure 5A). RNA sequencing identified 1009 DEGs in the AGEs group relative to controls, including 591 upregulated and 418 downregulated genes (Figure 5B). GSEA demonstrated significant enrichment of pathways associated with cellular senescence and inflammation, notably NF‐κB signaling and complement activation (Figure 5C). Consistently, expression profiling demonstrated downregulation of the cell cycle regulator Cdk1 and proliferation marker Pcna, alongside upregulation of Cdkn1a and Cdkn2a, indicating cell cycle arrest. Inflammatory mediators (IL‐6, Cxcl1, and CCL2) and matrix degradation marker Mmp13 were significantly increased, whereas cartilage‐specific genes Acan and Col2a1 were decreased. Elevated Bax expression further suggested enhanced apoptotic signaling (Figure 5D).

Proteomic analysis identified 24 differentially expressed proteins (DEPs) (Figure 5E). Protein‐level validation confirmed consistent alterations in cell cycle, inflammatory, and matrix‐related pathways (Figure 5F). Detailed GO/KEGG enrichment results, along with PPI network analyses, are provided in Figure S7.

Collectively, these multi‐omics findings indicate that AGE‐treated osteoclasts induce transcriptional and proteomic reprogramming toward a senescence‐associated phenotype in chondrocytes.

### 3.6 AGEs Induce Chondrocyte Senescence via the RAGE‐SIRT1‐RANKL/RANK Axis

**FIGURE 6:**
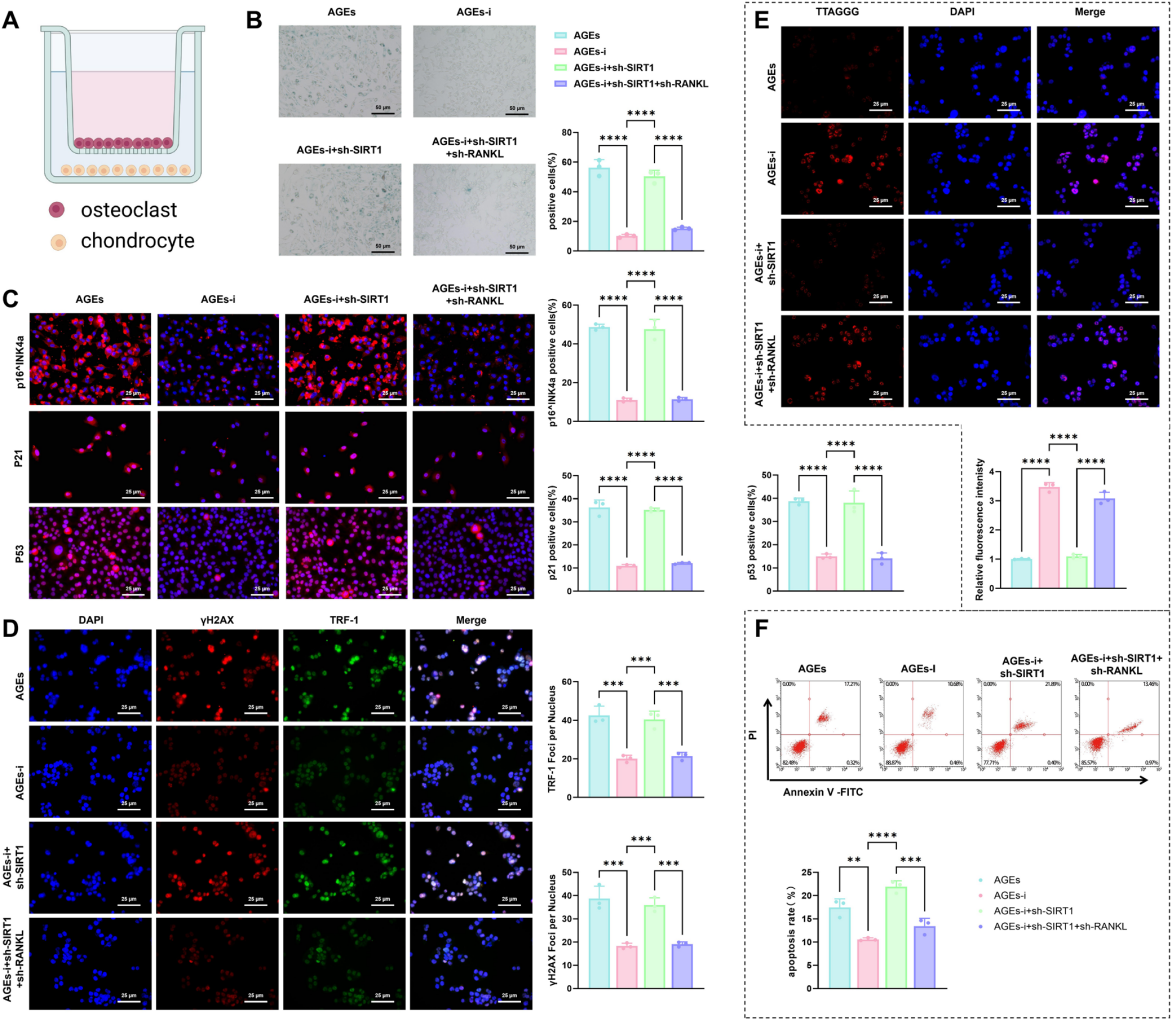
Effects of AGEs on chondrocyte senescence via the SIRT1/RANKL/RANK signaling pathway. (A) Schematic diagram of experimental grouping and treatment strategy; (B) SA‐β‐gal staining to assess the proportion of senescent chondrocytes in AGEs, AGEs‐i, AGEs‐i+sh‐SIRT1, and AGEs‐i+sh‐SIRT1+sh‐RANKL groups, bar = 50 μm; (C) IF analysis of senescence markers p16^INK4a^, p21, and p53 in chondrocytes from the indicated groups, bar = 25 μm; (D) IF analysis of DNA damage marker γH2AX and telomere‐associated protein TRF‐1 in chondrocytes, bar = 25 μm; (E) Representative images from telomere FISH analysis, bar = 25 μm; (F) Flow cytometric analysis of apoptosis rates in chondrocytes from the indicated groups. All experiments were repeated in triplicate. *Indicates comparison between two groups; *p < 0.05, **p < 0.01, ***p < 0.001, ****p < 0.0001.

To investigate whether AGEs regulate chondrocyte senescence through the SIRT1/RANKL/RANK pathway in osteoclasts, osteoclasts from the AGEs, AGEs‐i, AGEs‐i+sh‐SIRT1, and AGEs‐i+sh‐SIRT1+sh‐RANKL groups were co‐cultured with chondrocytes using a Transwell system (Figure 6A). Chondrocyte senescence phenotypes, DNA damage, oxidative stress, telomere length, proliferation, and apoptosis were evaluated using SA‐β‐gal staining, immunofluorescence, ROS assays, telomere FISH, CCK‐8, and flow cytometry.

SA‐β‐gal staining demonstrated that the percentage of senescent chondrocytes was significantly lower in the AGEs‐i group than in the AGEs group. SIRT1 knockdown markedly increased SA‐β‐gal positivity in the AGEs‐i+sh‐SIRT1 group, whereas additional RANKL silencing reduced it again in the AGEs‐i+sh‐SIRT1+sh‐RANKL group (Figure 6B). IF analysis showed that the expression of senescence markers p16^INK4a^, P21, and P53 (Figure 6C), as well as SASP factors TNF‐α, IL‐6, and IL‐1β (Figure S8A), was substantially decreased following RAGE inhibition. These markers were significantly elevated after SIRT1 silencing and subsequently diminished upon RANKL knockdown (Figure 6C and Figure S7B). These findings indicate that RAGE inhibition suppresses chondrocyte senescence by restoring SIRT1 expression, while SIRT1 silencing reverses this effect.

Consistent with the senescence phenotype, γH2AX and TRF‐1 expression in chondrocytes was significantly reduced in the AGEs‐i group compared with the AGEs group (Figure 6D), along with increased telomere length (Figure 6E). SIRT1 knockdown reversed these effects, as evidenced by increased γH2AX and TRF‐1 levels and shortened telomeres in the AGEs‐i+sh‐SIRT1 group relative to AGEs‐i (Figure 6D,E). Notably, additional RANKL silencing attenuated these changes, leading to decreased marker expression and restoration of telomere length in the AGEs‐i+sh‐SIRT1+sh‐RANKL group (Figure 6D,E). ROS analysis further revealed that ROS activity was significantly decreased in the AGEs‐i group, elevated in the AGEs‐i+sh‐SIRT1 group, and again reduced in the AGEs‐i+sh‐SIRT1+sh‐RANKL group (Figure S8B). These results suggest that RAGE blockade alleviates DNA damage and oxidative stress by restoring SIRT1 expression; this effect is reversed by SIRT1 silencing, while RANKL knockdown further attenuates DNA damage and oxidative stress.

CCK‐8 analysis demonstrated that chondrocyte proliferation increased significantly in the AGEs‐i group relative to the AGEs group, declined markedly after SIRT1 silencing in the AGEs‐i+sh‐SIRT1 group, and was partially restored upon additional RANKL knockdown in the AGEs‐i+sh‐SIRT1+sh‐RANKL group (Figure S8C). Flow cytometric analysis showed a consistent pattern: apoptosis was markedly reduced in the AGEs‐i group, increased following SIRT1 knockdown, and subsequently diminished again after additional RANKL silencing (Figure 6F). These results indicate that the RAGE inhibitor promotes chondrocyte proliferation and inhibits apoptosis by restoring SIRT1 expression, whereas SIRT1 silencing reverses these effects. Notably, RANKL silencing further enhances proliferation and suppresses apoptosis.

Collectively, these findings suggest that AGEs regulate osteoclast‐mediated chondrocyte senescence through the RAGE‐SIRT1‐RANKL/RANK signaling axis.

### 3.7 AGEs Promote OA Progression in Mice via the RAGE‐SIRT1‐RANKL/RANK Axis

**FIGURE 7:**
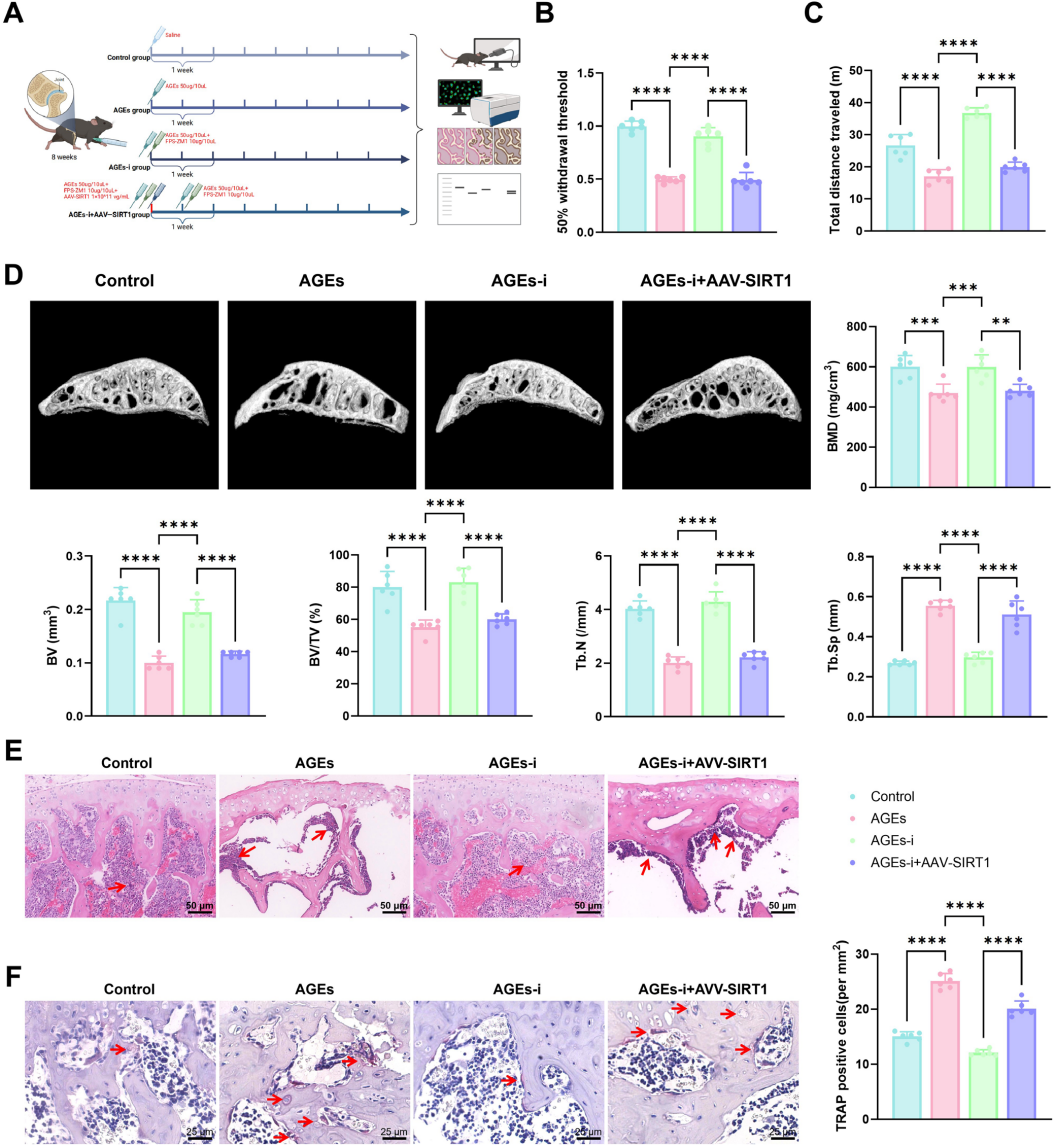
In vivo validation of the AGEs/SIRT1/RANKL/RANK signaling pathway in OA progression. (A) Schematic diagram of the animal experimental design and treatment protocol; (B) Assessment of joint pain sensitivity in control, AGEs, AGEs‐i, and AGEs‐i+AAV‐SIRT1 groups using electronic algometry; (C) Behavioral analysis of locomotor activity in the four groups; (D) Micro‐CT analysis of BMD, BV, BV/TV, Tb.N, and Tb.Sp; (E) H&E staining to evaluate joint inflammation, red arrows indicate representative histological features, bar = 50 μm; (F) TRAP staining to assess the number of osteoclasts in joint tissues, red arrows indicate representative positive cells, bar = 25 μm. *n* = 6 mice per group. *Indicates comparison between groups; **p < 0.01, ****p < 0.0001.

To investigate the role of the AGEs/SIRT1/RANKL/RANK signaling pathway in OA progression, a collagenase‐induced OA model was generated in 8‐week‐old male C57BL/6 mice. Animals were randomly allocated into four groups: Control, AGEs, AGEs‐i, and AGEs‐i+AAV‐SIRT1 (Figure 7A). Joint function, bone structure, cartilage damage, and senescence phenotype were assessed through behavioral tests, imaging analyses, histological staining, and molecular biology techniques.

Pain sensitivity analysis demonstrated that mice in the AGEs group displayed significantly heightened joint nociception compared with the Control group. RAGE inhibition markedly alleviated pain responses in the AGEs‐i group, whereas pain sensitivity increased again following AAV‐SIRT1 intervention in the AGEs‐i+AAV‐SIRT1 group (Figure 7B). Locomotor activity assessment revealed a pronounced decline in locomotor activity in the AGEs group. Movement distance was improved in the AGEs‐i group but was reduced again in the AGEs‐i+AAV‐SIRT1 group (Figure 7C). These results suggest that the RAGE–SIRT1–RANKL/RANK axis significantly affects joint function.

Micro‐CT analysis demonstrated that, compared with controls, mice in the AGEs group showed markedly decreased BMD, BV, BV/TV, and trabecular number (Tb.N), accompanied by a significant increase in trabecular separation (Tb.Sp). These structural impairments were substantially alleviated in the AGEs‐i group but worsened again following AAV‐SIRT1 treatment in the AGEs‐i+AAV‐SIRT1 group (Figure 7D). H&E staining revealed pronounced inflammation in joint tissues of the AGEs group, which was alleviated in the AGEs‐i group and exacerbated in the AGEs‐i+AAV‐SIRT1 group (Figure 7E). TRAP staining showed a significant increase in osteoclast number in the AGEs group, a reduction in the AGEs‐i group, and a re‐elevation in the AGEs‐i+AAV‐SIRT1 group (Figure 7F).

**FIGURE 8:**
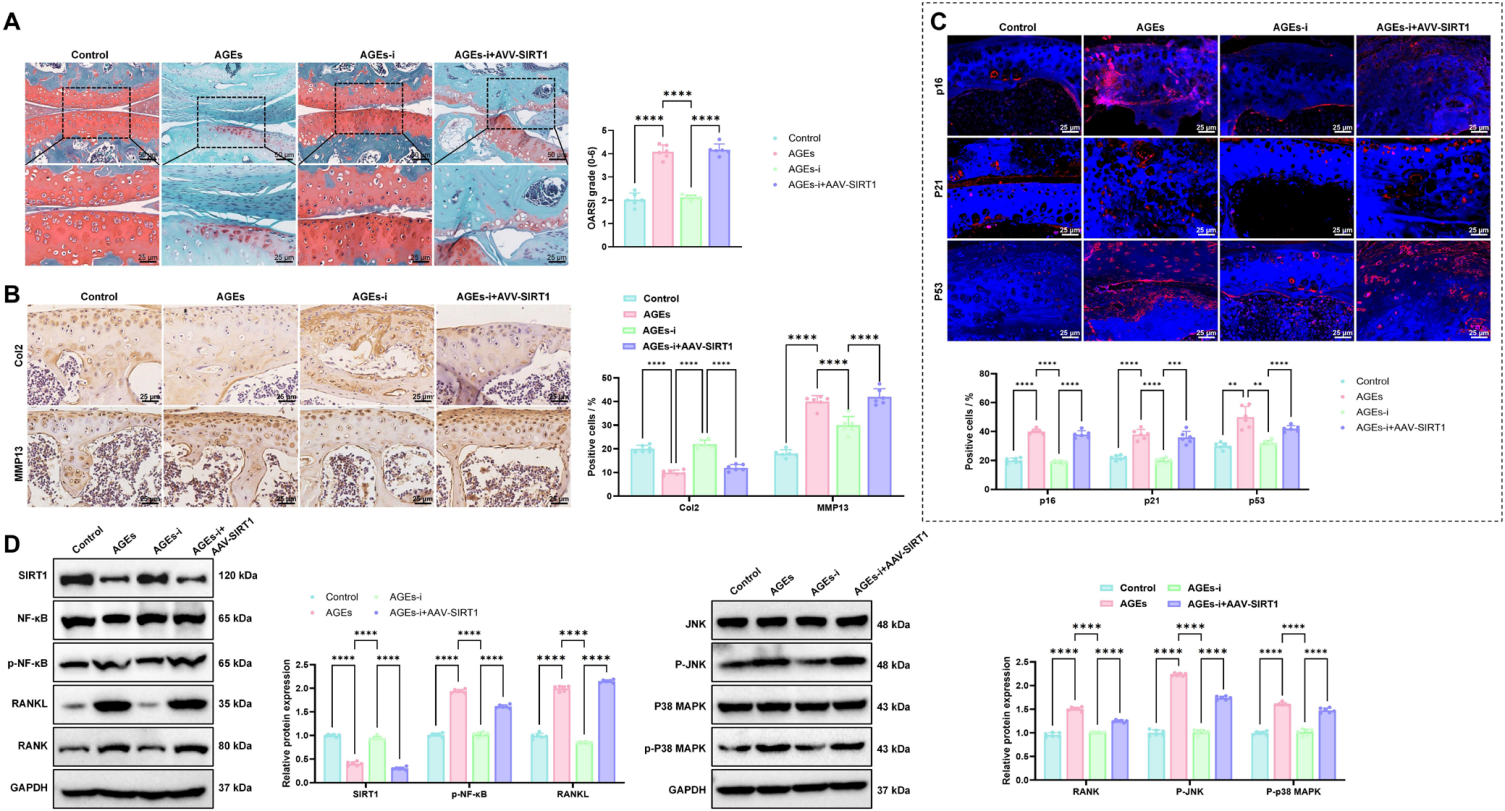
In vivo validation of the effects of AGEs/SIRT1/RANKL/RANK signaling on cartilage damage and senescence. (A) Safranin O‐Fast Green staining to evaluate cartilage damage and proteoglycan content in joint tissues, bar = 25 μm; (B) IHC analysis of Col2 and MMP13 expression, bar = 25 μm; (C) IF analysis of senescence markers p16^INK4a^, p21, and p53 in chondrocytes, bar = 25 μm; (D) Western blot analysis of SIRT1, RANKL, RANK, and downstream signaling molecules NF‐κB, JNK, and p38 MAPK and their phosphorylation levels in joint tissues. *n* = 6 mice per group. *Indicates comparison between groups; *p < 0.05, **p < 0.01, ***p < 0.001, ****p < 0.0001.

Safranin O‐Fast Green staining revealed that cartilage damage was significantly aggravated and proteoglycan content markedly reduced in the AGEs group compared with the Control group. In the AGEs‐i group, cartilage damage was alleviated and proteoglycan content restored, whereas in the AGEs‐i+AAV‐SIRT1 group, cartilage damage worsened and proteoglycan content declined again (Figure 8A). IHC staining showed that, compared to the Control group, the proportion of Col2‐positive chondrocytes (a cartilage matrix synthesis marker) was significantly decreased, while MMP13‐positive cells (a matrix degradation marker) were significantly increased in the AGEs group. In the AGEs‐i group, the proportion of Col2‐positive chondrocytes was increased, whereas MMP13‐positive cells were reduced. These changes were reversed following AAV‐SIRT1 intervention, as evidenced by decreased Col2 positivity and elevated MMP13 expression in the AGEs‐i+AAV‐SIRT1 group (Figure 8B). IF analysis demonstrated that the expression of senescence markers (p16^INK4a^, P21, and P53; Figure 8C) and SASP factors (TNF‐α, IL‐6, and IL‐1β; Figure S9A) were markedly increased in the AGEs group, significantly suppressed in the AGEs‐i group, and elevated again in the AGEs‐i + AAV‐SIRT1 group (Figure 8C and Figure S8A). WB analysis further confirmed that SIRT1 expression was markedly suppressed in the AGEs group, while the phosphorylation levels of RANKL, RANK, and downstream signaling molecules, including NF‐κB, JNK, and p38 MAPK, were significantly elevated. These effects were reversed in the AGEs‐i group, as evidenced by restored SIRT1 expression and reduced phosphorylation levels of RANKL/RANK pathway components. In the AGEs‐i+AAV‐SIRT1 group, SIRT1 expression was again decreased, with reactivation of the RANKL/RANK signaling cascade (Figure 8D).

Collectively, these results indicate that AGEs accelerate OA progression by promoting joint inflammation, subchondral bone loss, cartilage degradation, and chondrocyte senescence, primarily through activation of the RAGE–SIRT1–RANKL/RANK signaling axis.

## 4 Discussion

AGEs, as critical mediators in metabolic disorders, have garnered increasing attention for their pathogenic role in OA (He et al. 2022; Yang et al. 2022). While previous studies have primarily focused on AGE‐induced oxidative stress and matrix degradation directly within chondrocytes (Huang et al. 2019), investigations into their regulatory role in intercellular communication within the joint microenvironment remain limited (Chen et al. 2018; Xie et al. 2013). In the present study, we shift the perspective toward osteoclast‐mediated signaling, demonstrating that AGEs profoundly alter osteoclast biology via the RAGE–SIRT1–RANKL axis, which secondarily drives chondrocyte senescence through paracrine crosstalk. This finding challenges the traditional cartilage‐centric view by establishing a “metabolic–epigenetic–signaling” cascade where bone‐derived signals actively propagate joint degeneration. Specifically, AGEs suppress SIRT1 expression via RAGE, leading to epigenetic derepression of the Tnfsf11 (RANKL) promoter and subsequent activation of RANKL/RANK signaling. This axis effectively links metabolic stress to dysregulated bone remodeling and accelerated cartilage aging.

SIRT1, a well‐characterized deacetylase, exerts anti‐inflammatory, antioxidative, and anti‐senescent effects across various tissues (Cai et al. 2023). In OA research, its protective functions have been predominantly described in chondrocytes, where it suppresses p53 and NF‐κB signaling to delay senescence and apoptosis (Shakibaei et al. 2011; Yan et al. 2019). Our findings expand this framework by identifying osteoclasts as an additional critical cellular target of SIRT1 regulation. Importantly, mechanistic analysis in this study clarifies that SIRT1 does not directly deacetylate the RANKL protein but instead functions as a histone deacetylase at the Tnfsf11 (RANKL) promoter. AGE‐induced loss of SIRT1 leads to hyperacetylation of histone H3 (H3K27ac) at this locus, creating an open chromatin state that facilitates RANKL transcription. Furthermore, although AGE accumulation often coincides with broad metabolic stress, pharmacological validation using the specific inhibitor FPS‐ZM1 confirms that this pathological cascade is primarily initiated through AGE–RAGE receptor engagement. Blocking RAGE significantly restored SIRT1 expression and suppressed RANKL activation, suggesting that receptor‐mediated signaling constitutes the upstream trigger, distinguishing this pathway from nonspecific cellular oxidative stress responses.

The RANKL/RANK signaling pathway is a critical regulatory axis for osteoclast differentiation during bone remodeling and is widely studied in osteoporosis research. However, its involvement in OA remains relatively underexplored (Liu et al. 2010; Cao 2018; Yasuda 2021). Historically, osteoclasts and chondrocytes have been considered functionally independent, with osteoclasts primarily responsible for bone metabolism and chondrocytes for cartilage homeostasis (Takeshita et al. 2021). However, emerging evidence suggests a high degree of coupling between these two cell types in joint degenerative diseases (Guo et al. 2022; Dai et al. 2024; Jiang et al. 2023). By establishing an osteoclast–chondrocyte co‐culture system, this study delineates the upstream AGEs–SIRT1–RANKL regulatory axis driving this coupling. Beyond the classical RANKL‐dependent mechanism, secretome profiling further demonstrated that AGE‐stimulated osteoclasts acquire a pronounced pro‐inflammatory phenotype, characterized by elevated secretion of clastokines such as IL‐6 and TNF‐α. Neutralization experiments demonstrated that blockade of these cytokines partially attenuated chondrocyte senescence, indicating that inflammatory mediators act as cooperative amplifiers in the joint microenvironment. However, the comparatively weaker rescue effect relative to direct RANKL inhibition suggests a hierarchical organization. In this model of metabolic OA, the SIRT1–RANKL axis appears to function as the dominant driver of osteoclast‐induced chondrocyte aging, with inflammatory cytokines playing a supportive role.

In this study, a combined proteomic and transcriptomic strategy was applied to systematically characterize the molecular alterations in osteoclasts upon stimulation with AGEs. A machine learning algorithm (LASSO regression) was introduced for feature extraction and core target identification across multi‐omics datasets (Sanches et al. 2024; Clark et al. 2023). This rigorous screening process identified SIRT1 as a key node, significantly downregulated at both the protein and mRNA levels, and centrally positioned within the regulatory network. This integrative strategy offers a novel framework for dissecting complex signaling pathways, particularly in chronic diseases involving multicellular interactions such as OA, and holds translational potential for application in other degenerative disorders (Kumar et al. 2024; Li et al. 2022).

To validate the functional relevance of the AGEs–SIRT1–RANKL pathway in vivo, we established a murine OA model and implemented targeted pharmacological interventions. The findings showed that AGEs markedly aggravated cartilage degradation and promoted osteoclast activation in vivo. In contrast, activation of SIRT1 or suppression of RANKL substantially alleviated these pathological alterations, aligning closely with the in vitro results. Notably, in the context of metabolic or age‐related OA, characterized by elevated systemic levels of AGEs, the activation of this pathway likely holds high clinical relevance. These in vivo data not only validate the proposed mechanism but also provide a solid theoretical foundation and translational basis for future drug development and subtype‐specific interventions in OA (Park et al. 2020). Current clinical management of OA relies predominantly on symptomatic relief. By approaching OA from the perspective of metabolic dysregulation, this study identifies SIRT1 as a pivotal node mediating bone‐cartilage crosstalk, offering a systemic therapeutic target. Given that SIRT1 activators and RAGE inhibitors have demonstrated favorable safety profiles in other contexts, these findings provide a realistic foundation for clinical translation.

Despite these compelling findings, extrapolating murine data to human OA pathology requires caution. First, substantial differences exist in joint biomechanics; the quadrupedal loading pattern in mice differs significantly from the bipedal weight‐bearing mechanics in humans, which may influence localized mechanosensitive signaling. Second, there are profound distinctions in metabolic profiles and aging timelines. Humans accumulate AGEs over decades of chronic metabolic stress, whereas our model relies on acute induction over a relatively short period. Consequently, the rapid onset of phenotypes in mice may not fully capture the gradual, cumulative nature of chondrocyte aging in elderly OA patients. Third, laboratory mice possess a uniform genetic background, whereas human OA is characterized by high heterogeneity. Nevertheless, given the evolutionary conservation of the AGE‐RAGE axis and SIRT1 signaling across mammals, our findings offer a valid mechanistic framework. Future studies should focus on validating this pathway using human OA specimens (e.g., osteoclasts or synovial fluid) and further exploring the crosstalk between AGEs and other aging‐related pathways.

In conclusion, this study identifies a critical pathogenic cascade where AGEs downregulate SIRT1 to epigenetically activate RANKL in osteoclasts, subsequently inducing chondrocyte senescence via paracrine signaling. This work not only deepens the understanding of metabolic OA but also highlights the therapeutic promise of targeting upstream metabolic and epigenetic regulators in the joint microenvironment.

## 5 Conclusion

This study reveals that AGEs promote OA progression by suppressing SIRT1 expression and activity, inhibiting deacetylation of the RANKL promoter, upregulating RANKL expression, and activating the RANKL/RANK signaling pathway, thereby enhancing osteoclast differentiation and chondrocyte senescence. In vitro experiments demonstrated that AGEs downregulate SIRT1 via RAGE‐mediated signaling, activating RANKL/RANK and its downstream effectors, including NF‐κB, JNK, and p38 MAPK, which in turn induce osteoclast differentiation and chondrocyte aging. In vivo experiments further confirmed that AGEs exacerbate joint inflammation, bone destruction, and chondrocyte senescence in mice. In contrast, pharmacological inhibition of RAGE or silencing of SIRT1 significantly attenuates these pathological changes. These findings elucidate a central role of AGEs in OA pathogenesis and highlight the SIRT1/RANKL/RANK signaling axis as a critical regulatory mechanism mediating osteoclast–chondrocyte communication.

This work provides novel molecular insights into the pathophysiology of OA and identifies the AGEs‐SIRT1‐RANKL/RANK axis as a key contributor to disease progression. These findings establish a mechanistic basis for targeted interventions, including RAGE inhibition and SIRT1 activation, as potential strategies to slow OA progression. In addition, the identified signaling components may serve as promising biomarkers and therapeutic targets for early diagnosis and precision treatment of OA.

## Author Contributions

Yizhou Li and Jian Wu contributed equally to this work. Yizhou Li conceived the research idea and designed the overall study. Jian Wu conducted the experiments and performed the data analysis. Rui Liu assisted with methodology development and contributed to data curation. Qiang Li provided technical guidance and supported the interpretation of results. Fei Xue supervised the project, contributed to the study design, and revised the manuscript. All authors discussed the results, contributed to the manuscript writing, and approved the final version of the paper.

## Funding

This study was supported by the Science and Technology Program Project of Inner Mongolia Autonomous Region (No. 2025YFSH0111), Public Hospital Joint Research Fund Science and Technology Project (Grant No. 2025GLLH0126), and Natural Science Foundation of Inner Mongolia Autonomous Region (Grant No. 2025LHMS08054).

## Ethics Statement

All animal experiments were approved by the Animal Ethics Committee of The Second Affiliated Hospital of Inner Mongolia Medical University (No. Y.2025130).

## Conflicts of Interest

The authors declare no conflicts of interest.

## Supporting information


**Figure S1:** Effects of AGEs on osteoclast differentiation. (A) GO and KEGG pathway enrichment analysis of DEGs from RNA‐seq data; (B) LASSO coefficient distribution of DEGs for feature selection; (C) Selection of the optimal lambda value in the LASSO regression model via cross‐validation; (D) GO and KEGG enrichment analysis of DEPs from proteomic data; (E) Schematic diagram of osteoclast precursor cell culture and differentiation; (F) F‐actin ring staining to evaluate cytoskeletal reorganization in osteoclasts, bar = 50 μm; (G) TRAP staining to assess osteoclast differentiation, bar = 50 μm. Experiments were repeated three times. *Indicates comparison between groups; ***p < 0.001, ****p < 0.0001.

**Figure S2:** GSEA analysis of AGE‐induced osteoclast differentiation. (A) GSEA enrichment analysis of DEGs from transcriptomic sequencing; (B) GSEA enrichment analysis of DEPs from proteomic sequencing.

**Figure S3:** Effects of AGEs on SIRT1 expression and RANKL/RANK signaling. (A) RT‐qPCR analysis of SIRT1 mRNA expression in control and AGEs groups; (B) Western blot analysis of SIRT1 protein expression; (C) SIRT1 activity assay; (D) RT‐qPCR analysis of RANKL and RANK mRNA levels in control and AGEs groups; (E) SIRT1 activity assay in AGEs and AGEs‐i groups; (F) RT‐qPCR analysis of RANKL and RANK mRNA expression in AGEs and AGEs‐i groups. Experiments were repeated three times. *Indicates comparison between groups; **p < 0.01, ***p < 0.001, ****p < 0.0001.

**Figure S4:** Effects of SIRT1 and RANKL silencing on AGEs‐induced osteoclast differentiation. (A) RT‐qPCR analysis of SIRT1 silencing efficiency; (B) Western blot analysis of SIRT1 silencing efficiency; (C) RT‐qPCR analysis of SIRT1, RANKL, and RANK mRNA levels in AGEs‐i and AGEs‐i+sh‐SIRT1 groups; (D) RT‐qPCR analysis of RANKL silencing efficiency; (E) Western blot analysis of RANKL silencing efficiency; (F) RT‐qPCR analysis of SIRT1, RANKL, and RANK mRNA expression in AGEs‐i+sh‐SIRT1 and AGEs‐i+sh‐SIRT1+sh‐RANKL groups. Experiments were repeated three times. *Indicates comparison between groups; ns, *p* > 0.05, **p < 0.01, ***p < 0.001, ****p < 0.0001.

**Figure S5:** Effects of AGE‐treated osteoclasts on chondrocyte SASP and cellular functions. (A) IF analysis of SASP markers TNF‐α, IL‐6, and IL‐1β in chondrocytes from control and AGEs groups, bar = 25 μm; (B) ROS assay for detecting ROS activity in chondrocytes, bar = 25 μm; (C) CCK‐8 assay for evaluating chondrocyte proliferation in control and AGEs groups. Experiments were repeated three times. *Indicates comparison between groups; **p < 0.01, ***p < 0.001, ****p < 0.0001.

**Figure S6:** Analysis of osteoclast‐derived paracrine mediators and their contribution to chondrocyte senescence. (A–D) ELISA quantification of IL‐6, TNF‐α, IL‐1β, and soluble RANKL (sRANKL) levels in the conditioned medium of osteoclasts (OC‐CM) from Control and AGEs groups. (E) Representative SA‐β‐gal staining images of chondrocytes treated with AGEs‐OC‐CM. Scale bar: 50 μm. Data are presented as mean ± SD from three independent experiments. *p < 0.05, **p < 0.01 versus Control‐CM group; #*p* < 0.05, ##*p* < 0.01 versus AGEs‐CM + IgG group.

**Figure S7:** Extended enrichment and network analyses of transcriptomic and proteomic alterations. (A) GO enrichment analysis of RNA‐seq data; (B) KEGG pathway enrichment analysis of differentially expressed genes; (C) PPI network of differentially expressed proteins; (D) GO and KEGG enrichment analyses of differentially expressed proteins.

**Figure S8:** Effects of AGEs on chondrocyte SASP and function via the SIRT1/RANKL/RANK pathway. (A) IF analysis of SASP markers TNF‐α, IL‐6, and IL‐1β in chondrocytes from AGEs, AGEs‐i, AGEs‐i+sh‐SIRT1, and AGEs‐i+sh‐SIRT1+sh‐RANKL groups, bar = 25 μm; (B) ROS assay detecting ROS activity in chondrocytes from the same groups, bar = 25 μm; (C) CCK‐8 assay assessing chondrocyte proliferation in the same groups. Experiments were repeated three times. *Indicates comparison between groups; **p < 0.01, ***p < 0.001, ****p < 0.0001.

**Figure S9:** In vivo validation of AGEs/SIRT1/RANKL/RANK signaling in regulating chondrocyte SASP. IF analysis of SASP markers TNF‐α, IL‐6, and IL‐1β in chondrocytes from control, AGEs, AGEs‐i, and AGEs‐i+AAV‐SIRT1 groups, bar = 25 μm. Each group included six mice. *Indicates comparison between groups; **p < 0.01, ***p < 0.001, ****p < 0.0001.

**Table S1:** Sequence of RT‐qPCR.
**Table S2:** Primary antibody information.
**Table S3:** Sequence of shRNA.

## Data Availability

The data that support the findings of this study are available from the corresponding author upon reasonable request.

## References

[acel70515-bib-0001] Aksoy, P. , C. Escande , T. A. White , et al. 2006. “Regulation of SIRT 1 Mediated NAD Dependent Deacetylation: A Novel Role for the Multifunctional Enzyme CD38.” Biochemical and Biophysical Research Communications 349, no. 1: 353–359. 10.1016/j.bbrc.2006.08.066. 16935261

[acel70515-bib-0002] Cai, M. , Y. Chen , Y. Lin , et al. 2023. “SIRT1 Asn346 Sugar Chain Promoting Collagen Deacetylation Protective Effect on Osteoblasts Under Stress.” Biochemical and Biophysical Research Communications 682: 148–155. 10.1016/j.bbrc.2023.09.075. 37806254

[acel70515-bib-0003] Cao, X. 2018. “RANKL‐RANK Signaling Regulates Osteoblast Differentiation and Bone Formation.” Bone Research 6: 35. 10.1038/s41413-018-0040-9. 30510840 PMC6255775

[acel70515-bib-0004] Chen, J.‐H. , X. Lin , C. Bu , et al. 2018. “Role of Advanced Glycation End Products in Mobility and Considerations in Possible Dietary and Nutritional Intervention Strategies.” Nutrition & Metabolism 15: 72. 10.1186/s12986-018-0306-7. 30337945 PMC6180645

[acel70515-bib-0005] Clark, N. M. , B. Hurgobin , D. R. Kelley , et al. 2023. “A Practical Guide to Inferring Multi‐Omics Networks in Plant Systems.” Methods in Molecular Biology 2698: 233–257. 10.1007/978-1-0716-3354-0_15. 37682479 PMC12704241

[acel70515-bib-0006] Corica, D. , G. Pepe , and M. Currò . 2022. “Methods to Investigate Advanced Glycation End‐Product and Their Application in Clinical Practice.” Methods 203: 90–102. 10.1016/j.ymeth.2021.12.008. 34942356

[acel70515-bib-0007] Dai, J. , Z. Hu , F. Zeng , et al. 2024. “Osteoclast‐Derived Exosomal miR‐212‐3p Suppressed the Anabolism and Accelerated the Catabolism of Chondrocytes in Osteoarthritis by Targeting TGF‐β1/Smad2 Signaling.” Archives of Biochemistry and Biophysics 751: 109827. 10.1016/j.abb.2023.109827. 38000494

[acel70515-bib-0008] Ding, X. , H. U. Yun , L. U. Dan , et al. 2020. “Nan Fang Yi Ke Da Xue Xue Bao.” Journal of Southern Medical University 40, no. 4: 573–579. 10.12122/j.issn.1673-4254.2020.04.20. 32895130 PMC7225107

[acel70515-bib-0009] Guo, P. , A. Alhaskawi , S. Adel Abdo Moqbel , et al. 2025. “Recent Development of Mitochondrial Metabolism and Dysfunction in Osteoarthritis.” Frontiers in Pharmacology 16: 1538662. 10.3389/fphar.2025.1538662. 40017603 PMC11865096

[acel70515-bib-0010] Guo, Y. N. , S. J. Cui , Y. J. Tian , et al. 2022. “Chondrocyte Apoptosis in Temporomandibular Joint Osteoarthritis Promotes Bone Resorption by Enhancing Chemotaxis of Osteoclast Precursors.” Osteoarthritis and Cartilage 30, no. 8: 1140–1153. 10.1016/j.joca.2022.04.002. 35513247

[acel70515-bib-0011] He, C.‐P. , C. Chen , X.‐C. Jiang , et al. 2022. “The Role of AGEs in Pathogenesis of Cartilage Destruction in Osteoarthritis.” Bone & Joint Research 11, no. 5: 292–300. 10.1302/2046-3758.115.BJR-2021-0334.R1. 35549515 PMC9130677

[acel70515-bib-0012] Huang, H. , Z.‐j. Wang , H.‐b. Zhang , et al. 2019. “The Function of PPARγ/AMPK/SIRT‐1 Pathway in Inflammatory Response of Human Articular Chondrocytes Stimulated by Advanced Glycation End Products.” Biological & Pharmaceutical Bulletin 42, no. 8: 1303–1309. 10.1248/bpb.b19-00036. 31366866

[acel70515-bib-0013] Ikebuchi, Y. , S. Aoki , M. Honma , et al. 2018. “Coupling of Bone Resorption and Formation by RANKL Reverse Signalling.” Nature 561, no. 7722: 195–200. 10.1038/s41586-018-0482-7. 30185903

[acel70515-bib-0014] Jeong, K.‐Y. , and H. J. Lee . 2021. “Prevalence of Knee Osteoarthritis and Health‐Related Quality of Life in Stroke Patients Over 60 Years Old: A Cross‐Sectional Study Using Korean National Health and Nutrition Examination Survey V.” Annals of Geriatric Medicine and Research 25, no. 3: 178–186. 10.4235/agmr.21.0053. 34275255 PMC8497948

[acel70515-bib-0015] Ji, M.‐L. , H. Jiang , Z. Li , et al. 2022. “Sirt6 Attenuates Chondrocyte Senescence and Osteoarthritis Progression.” Nature Communications 13, no. 1: 7658. 10.1038/s41467-022-35424-w. PMC9741608 36496445

[acel70515-bib-0016] Jiang, A. , P. Xu , Z. Yang , et al. 2023. “Increased Sparc Release From Subchondral Osteoblasts Promotes Articular Chondrocyte Degeneration Under Estrogen Withdrawal.” Osteoarthritis and Cartilage 31, no. 1: 26–38. 10.1016/j.joca.2022.08.020. 36241137

[acel70515-bib-0017] Kuang, G. , X. Tan , X. Liu , et al. 2024. “The Role of Innate Immunity in Osteoarthritis and the Connotation of “Immune‐Joint” Axis: A Narrative Review.” Combinatorial Chemistry & High Throughput Screening 27, no. 15: 2170–2179. 10.2174/0113862073264389231101190637. 38243960

[acel70515-bib-0018] Kumar, R. , R. Ruhel , and A. J. van Wijnen . 2024. “Unlocking Biological Complexity: The Role of Machine Learning in Integrative Multi‐Omics.” Academia Biology 2, no. 4: acadbiol7428. 10.20935/acadbiol7428. PMC11741185 39830067

[acel70515-bib-0019] Leah, E. 2011. “Osteoarthritis: Chondrocyte Protection—SIRTified?” Nature Reviews Rheumatology 7, no. 1: 3. 10.1038/nrrheum.2010.203. 21260965

[acel70515-bib-0020] Li, X. , J. Ma , L. Leng , et al. 2022. “MoGCN: A Multi‐Omics Integration Method Based on Graph Convolutional Network for Cancer Subtype Analysis.” Frontiers in Genetics 13: 806842. 10.3389/fgene.2022.806842. 35186034 PMC8847688

[acel70515-bib-0021] Liao, Z. , X. Cai , Y. Zheng , et al. 2024. “Sirtuin 1 in Osteoarthritis: Perspectives on Regulating Glucose Metabolism.” Pharmacological Research 202: 107141. 10.1016/j.phrs.2024.107141. 38490314

[acel70515-bib-0022] Liu, C. , T. S. Walter , P. Huang , et al. 2010. “Structural and Functional Insights of RANKL‐RANK Interaction and Signaling.” Journal of Immunology (Baltimore, Md.: 1950) 184, no. 12: 6910–6919. 10.4049/jimmunol.0904033. 20483727

[acel70515-bib-0023] Liu, S. , G. Zhang , N. Li , et al. 2025. “The Interplay of Aging and PANoptosis in Osteoarthritis Pathogenesis: Implications for Novel Therapeutic Strategies.” Journal of Inflammation Research 18: 1951–1967. 10.2147/JIR.S489613. 39959642 PMC11829118

[acel70515-bib-0024] Park, D. R. , J. Kim , G. M. Kim , et al. 2020. “Osteoclast‐Associated Receptor Blockade Prevents Articular Cartilage Destruction via Chondrocyte Apoptosis Regulation.” Nature Communications 11, no. 1: 4343. 10.1038/s41467-020-18208-y. PMC7455568 32859940

[acel70515-bib-0025] Qu, B. , K. Gong , H. Yang , et al. 2018. “SIRT1 Suppresses High Glucose and Palmitate‐Induced Osteoclast Differentiation via Deacetylating p66Shc.” Molecular and Cellular Endocrinology 474: 97–104. 10.1016/j.mce.2018.02.015. 29486220

[acel70515-bib-0026] Reddy, V. P. , P. Aryal , and E. K. Darkwah . 2022. “Advanced Glycation End Products in Health and Disease.” Microorganisms 10, no. 9: 1848. 10.3390/microorganisms10091848. 36144449 PMC9501837

[acel70515-bib-0027] Sanches, P. H. G. , N. C. de Melo , A. M. Porcari , et al. 2024. “Integrating Molecular Perspectives: Strategies for Comprehensive Multi‐Omics Integrative Data Analysis and Machine Learning Applications in Transcriptomics, Proteomics, and Metabolomics.” Biology 13, no. 11: 848. 10.3390/biology13110848. 39596803 PMC11592251

[acel70515-bib-0028] Shakibaei, M. , C. Buhrmann , and A. Mobasheri . 2011. “Resveratrol‐Mediated SIRT‐1 Interactions With p300 Modulate Receptor Activator of NF‐kappaB Ligand (RANKL) Activation of NF‐kappaB Signaling and Inhibit Osteoclastogenesis in Bone‐Derived Cells.” Journal of Biological Chemistry 286, no. 13: 11492–11505. 10.1074/jbc.M110.198713. 21239502 PMC3064204

[acel70515-bib-0029] Shane Anderson, A. , and R. F. Loeser . 2010. “Why Is Osteoarthritis an Age‐Related Disease?” Best Practice & Research. Clinical Rheumatology 24, no. 1: 15–26. 10.1016/j.berh.2009.08.006. 20129196 PMC2818253

[acel70515-bib-0030] Takeshita, A. , K. Nishida , A. Yoshida , et al. 2021. “RANKL Expression in Chondrocytes and Its Promotion by Lymphotoxin‐α in the Course of Cartilage Destruction During Rheumatoid Arthritis.” PLoS One 16, no. 7: e0254268. 10.1371/journal.pone.0254268. 34234380 PMC8263262

[acel70515-bib-0031] Terauchi, K. , H. Kobayashi , K. Yatabe , et al. 2016. “The NAD‐Dependent Deacetylase Sirtuin‐1 Regulates the Expression of Osteogenic Transcriptional Activator Runt‐Related Transcription Factor 2 (Runx2) and Production of Matrix Metalloproteinase (MMP)‐13 in Chondrocytes in Osteoarthritis.” International Journal of Molecular Sciences 17, no. 7: 1019. 10.3390/ijms17071019. 27367673 PMC4964395

[acel70515-bib-0032] Velasquez, M. T. , and J. D. Katz . 2010. “Osteoarthritis: Another Component of Metabolic Syndrome?” Metabolic Syndrome and Related Disorders 8, no. 4: 295–305. 10.1089/met.2009.0110. 20367223

[acel70515-bib-0033] Vitorakis, N. , and C. Piperi . 2024. “Pivotal Role of AGE‐RAGE Axis in Brain Aging With Current Interventions.” Ageing Research Reviews 100: 102429. 10.1016/j.arr.2024.102429. 39032613

[acel70515-bib-0034] Wei, Y. , and L. Bai . 2016. “Recent Advances in the Understanding of Molecular Mechanisms of Cartilage Degeneration, Synovitis and Subchondral Bone Changes in Osteoarthritis.” Connective Tissue Research 57, no. 4: 245–261. 10.1080/03008207.2016.1177036. 27285430

[acel70515-bib-0035] Wu, Z. , K. Yuan , Q. Zhang , et al. 2022. “Antioxidant PDA‐PEG Nanoparticles Alleviate Early Osteoarthritis by Inhibiting Osteoclastogenesis and Angiogenesis in Subchondral Bone.” Journal of Nanobiotechnology 20, no. 1: 479. 10.1186/s12951-022-01697-y. 36384720 PMC9670483

[acel70515-bib-0036] Xiao, S.‐Q. , M. Cheng , L. Wang , et al. 2023. “The Role of Apoptosis in the Pathogenesis of Osteoarthritis.” International Orthopaedics 47, no. 8: 1895–1919. 10.1007/s00264-023-05847-1. 37294429

[acel70515-bib-0037] Xie, J. , J. D. Méndez , V. Méndez‐Valenzuela , and M. M. Aguilar‐Hernández . 2013. “Cellular Signalling of the Receptor for Advanced Glycation End Products (RAGE).” Cellular Signalling 25, no. 11: 2185–2197. 10.1016/j.cellsig.2013.06.013. 23838007

[acel70515-bib-0038] Yan, S. , L. Miao , Y. Lu , and L. Wang . 2019. “Sirtuin 1 Inhibits TNF‐α‐Mediated Osteoclastogenesis of Bone Marrow‐Derived Macrophages Through Both ROS Generation and TRPV1 Activation.” Molecular and Cellular Biochemistry 455, no. 1–2: 135–145. 10.1007/s11010-018-3477-7. 30456702

[acel70515-bib-0039] Yang, Q. , Y. Shi , T. Jin , B. Duan , and S. Wu . 2022. “Advanced Glycation End Products Induced Mitochondrial Dysfunction of Chondrocytes Through Repression of AMPKα‐SIRT1‐PGC‐1α Pathway.” Pharmacology 107, no. 5–6: 298–307. 10.1159/000521720. 35240662

[acel70515-bib-0040] Yao, Q. , X. Wu , C. Tao , et al. 2023. “Osteoarthritis: Pathogenic Signaling Pathways and Therapeutic Targets.” Signal Transduction and Targeted Therapy 8, no. 1: 56. 10.1038/s41392-023-01330-w. 36737426 PMC9898571

[acel70515-bib-0041] Yasuda, H. 2021. “Discovery of the RANKL/RANK/OPG System.” Journal of Bone and Mineral Metabolism 39, no. 1: 2–11. 10.1007/s00774-020-01175-1. 33389131

[acel70515-bib-0042] Zhang, M. , Y. Li , P. Rao , et al. 2018. “Blockade of Receptors of Advanced Glycation End Products Ameliorates Diabetic Osteogenesis of Adipose‐Derived Stem Cells Through DNA Methylation and Wnt Signalling Pathway.” Cell Proliferation 51, no. 5: e12471. 10.1111/cpr.12471. 30014569 PMC6528890

[acel70515-bib-0043] Zhou, M. , Y. Zhang , L. Shi , et al. 2024. “Activation and Modulation of the AGEs‐RAGE Axis: Implications for Inflammatory Pathologies and Therapeutic Interventions—A Review.” Pharmacological Research 206: 107282. 10.1016/j.phrs.2024.107282. 38914383

