# Supplementary figures and images for: SIRT1 Downregulation by Advanced Glycation End Products Activates RANKL‐Dependent Osteoclast Signaling and Drives Chondrocyte Senescence During Osteoarthritis Development
Source: Aging Cell. 2026 May 1;25(5):e70515. doi: 10.1111/acel.70515 (PMC13134948; doi:10.1111/acel.70515)

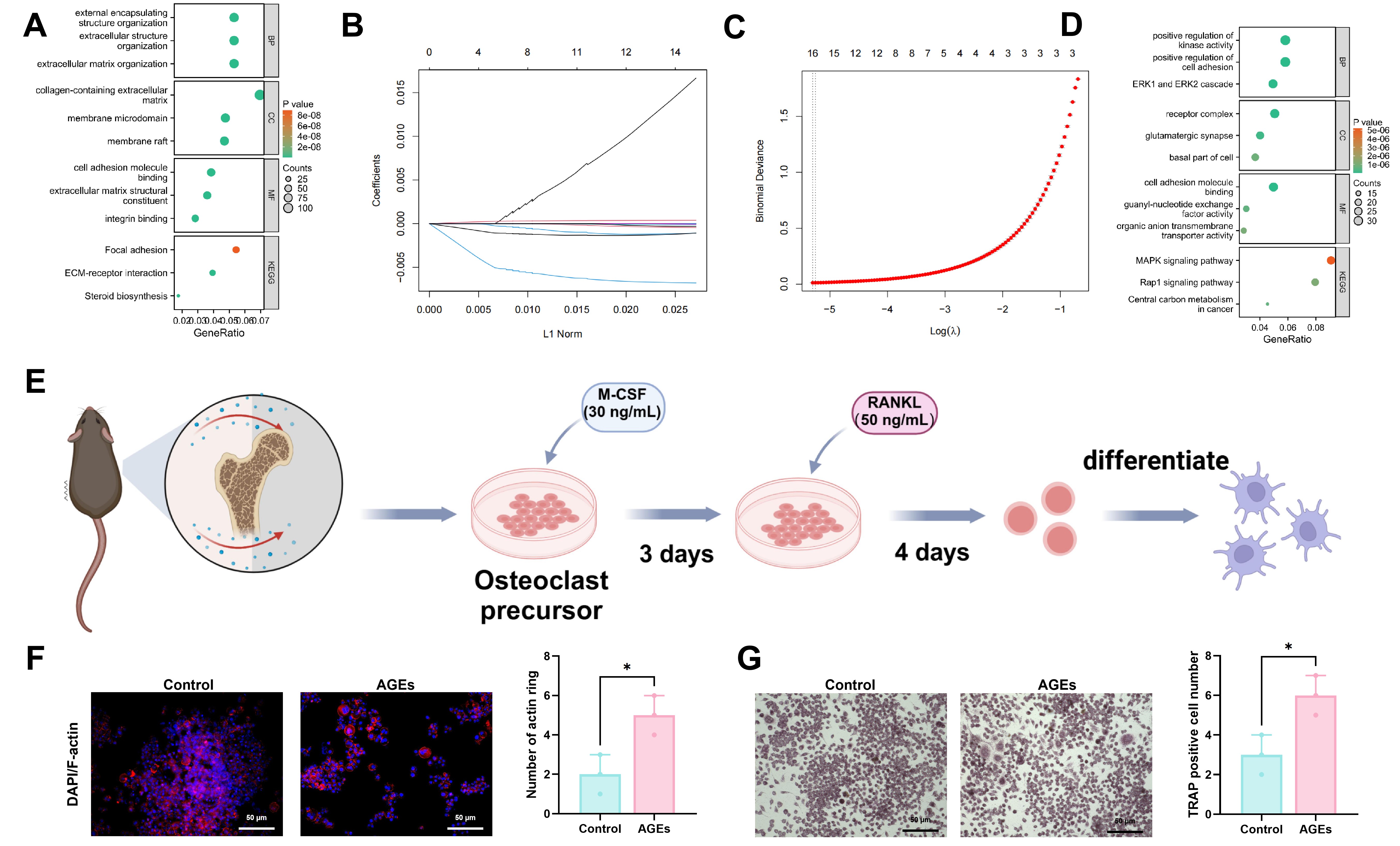

Supplement: Supplementary file 1 — Figure S1: Effects of AGEs on osteoclast differentiation. (A) GO and KEGG pathway enrichment analysis of DEGs from RNA‐seq data; (B) LASSO coefficient distribution of DEGs for feature selection; (C) Selection of the optimal lambda value in the LASSO regression model via cross‐validation; (D) GO and KEGG enrichment analysis of DEPs from proteomic data; (E) Schematic diagram of osteoclast precursor cell culture and differentiation; (F) F‐actin ring staining to evaluate cytoskeletal reorganization in osteoclasts, bar = 50 μm; (G) TRAP staining to assess osteoclast differentiation, bar = 50 μm. Experiments were repeated three times. *Indicates comparison between groups; ***p < 0.001, ****p < 0.0001. [file ACEL-25-e70515-s002.jpg]

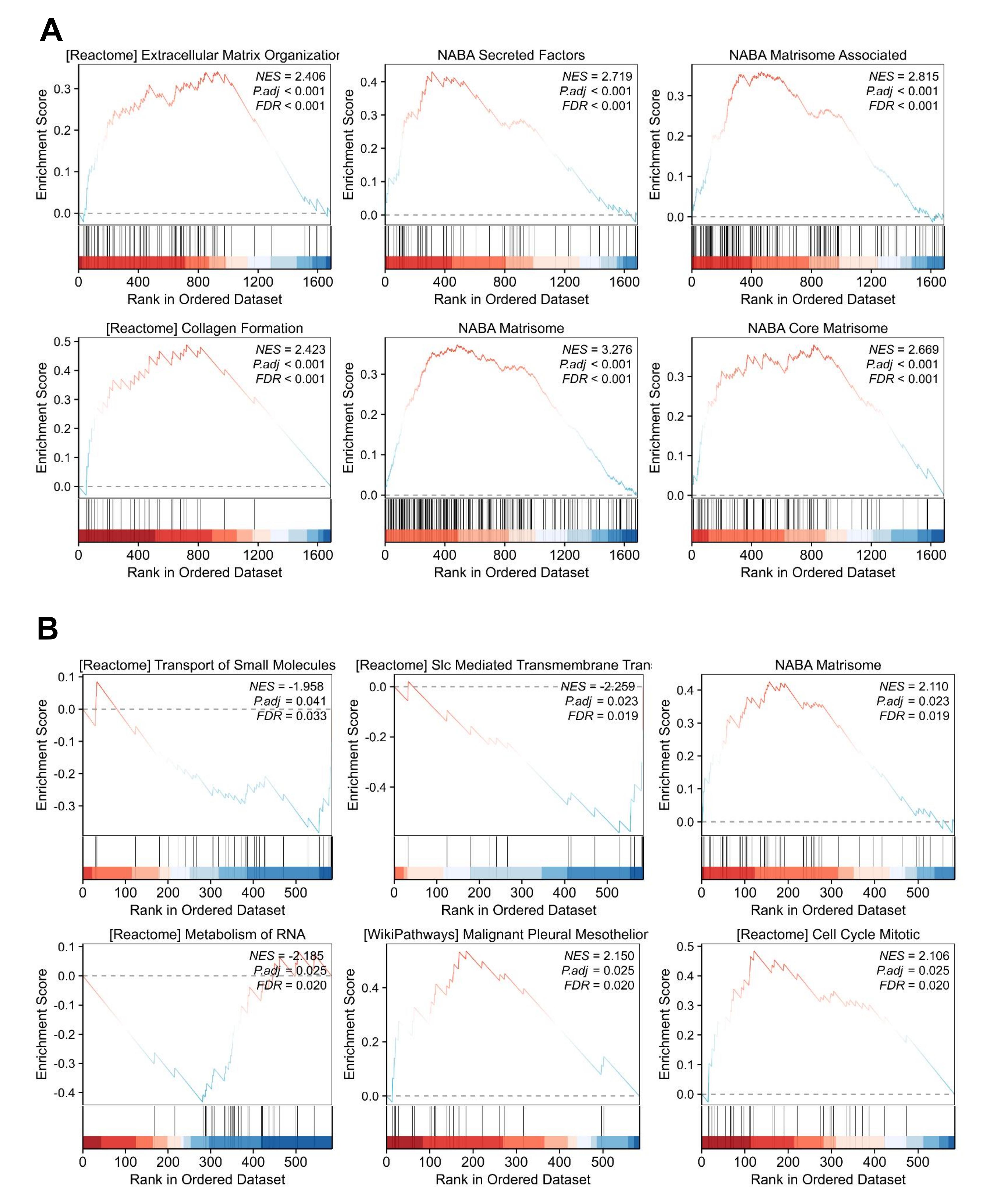

Supplement: Supplementary file 2 — Figure S2: GSEA analysis of AGE‐induced osteoclast differentiation. (A) GSEA enrichment analysis of DEGs from transcriptomic sequencing; (B) GSEA enrichment analysis of DEPs from proteomic sequencing. [file ACEL-25-e70515-s004.jpg]

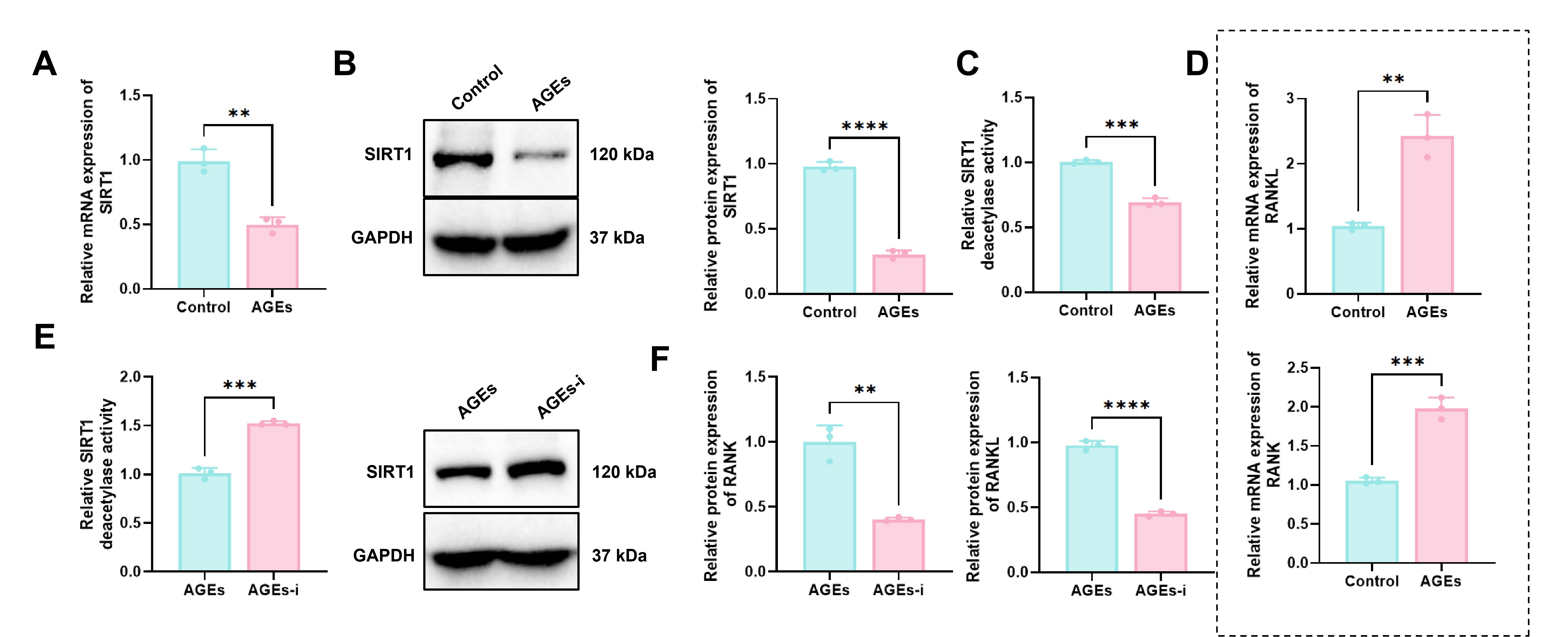

Supplement: Supplementary file 3 — Figure S3: Effects of AGEs on SIRT1 expression and RANKL/RANK signaling. (A) RT‐qPCR analysis of SIRT1 mRNA expression in control and AGEs groups; (B) Western blot analysis of SIRT1 protein expression; (C) SIRT1 activity assay; (D) RT‐qPCR analysis of RANKL and RANK mRNA levels in control and AGEs groups; (E) SIRT1 activity assay in AGEs and AGEs‐i groups; (F) RT‐qPCR analysis of RANKL and RANK mRNA expression in AGEs and AGEs‐i groups. Experiments were repeated three times. *Indicates comparison between groups; **p < 0.01, ***p < 0.001, ****p < 0.0001. [file ACEL-25-e70515-s008.jpg]

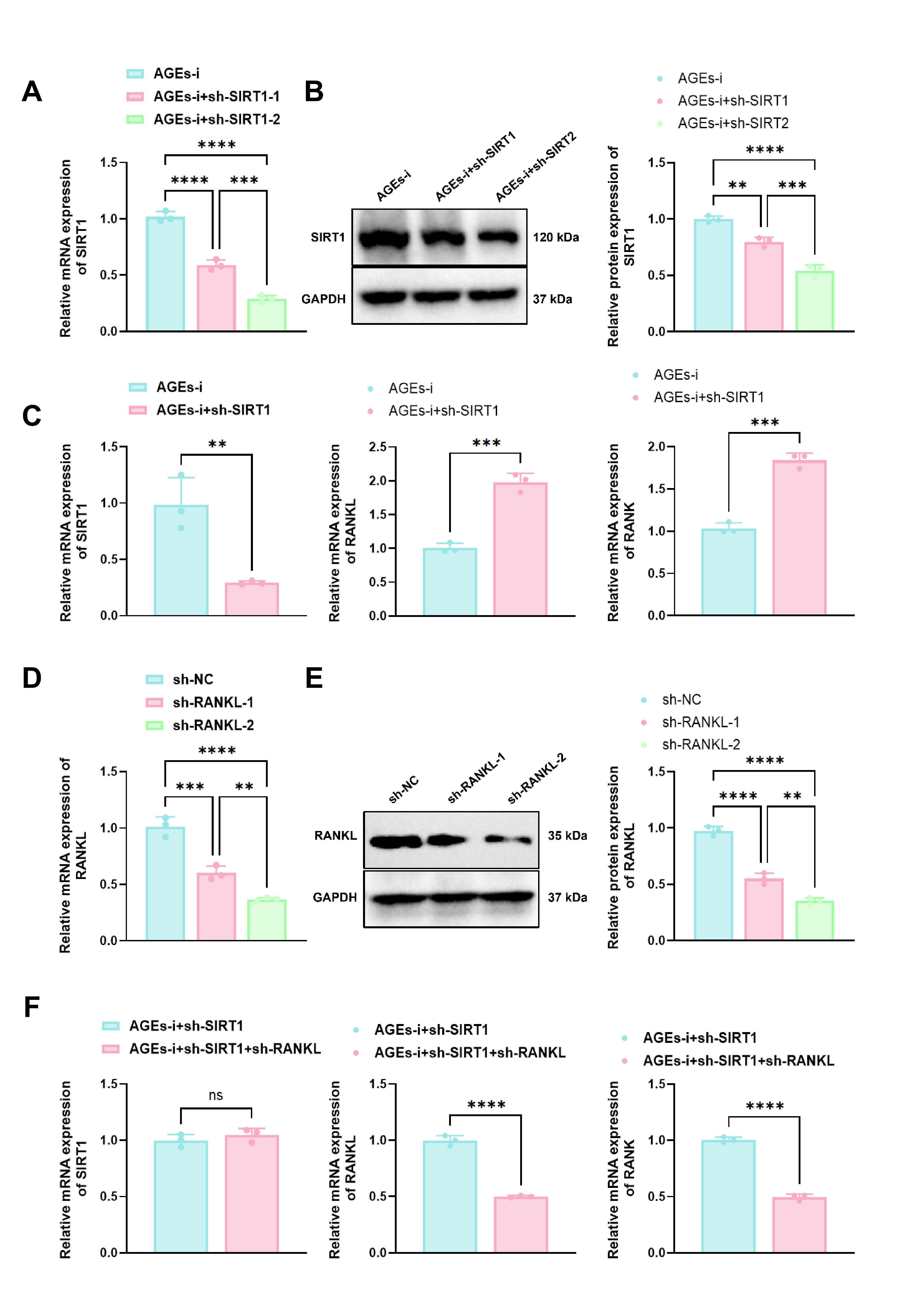

Supplement: Supplementary file 4 — Figure S4: Effects of SIRT1 and RANKL silencing on AGEs‐induced osteoclast differentiation. (A) RT‐qPCR analysis of SIRT1 silencing efficiency; (B) Western blot analysis of SIRT1 silencing efficiency; (C) RT‐qPCR analysis of SIRT1, RANKL, and RANK mRNA levels in AGEs‐i and AGEs‐i+sh‐SIRT1 groups; (D) RT‐qPCR analysis of RANKL silencing efficiency; (E) Western blot analysis of RANKL silencing efficiency; (F) RT‐qPCR analysis of SIRT1, RANKL, and RANK mRNA expression in AGEs‐i+sh‐SIRT1 and AGEs‐i+sh‐SIRT1+sh‐RANKL groups. Experiments were repeated three times. *Indicates comparison between groups; ns, p > 0.05, **p < 0.01, ***p < 0.001, ****p < 0.0001. [file ACEL-25-e70515-s009.jpg]

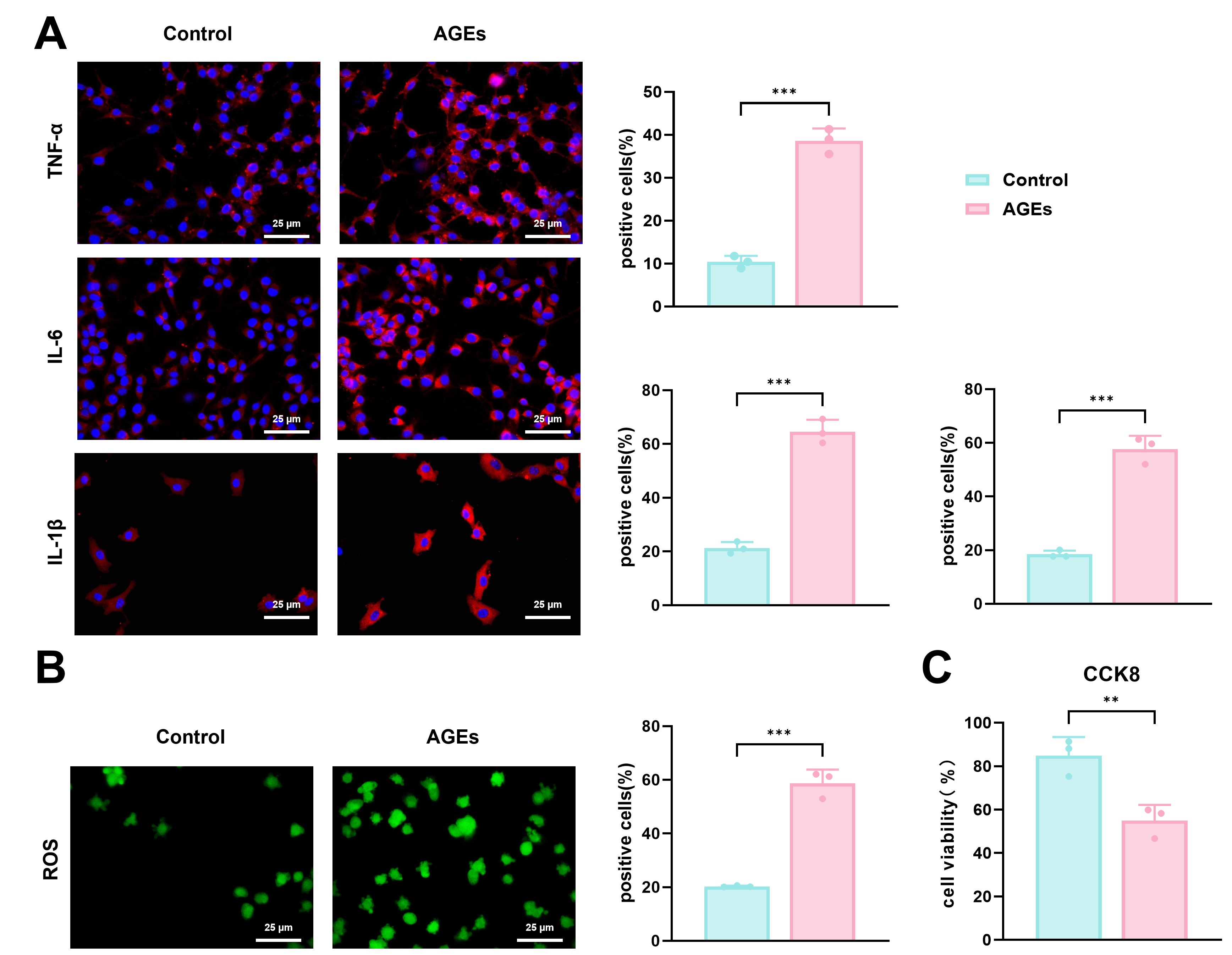

Supplement: Supplementary file 5 — Figure S5: Effects of AGE‐treated osteoclasts on chondrocyte SASP and cellular functions. (A) IF analysis of SASP markers TNF‐α, IL‐6, and IL‐1β in chondrocytes from control and AGEs groups, bar = 25 μm; (B) ROS assay for detecting ROS activity in chondrocytes, bar = 25 μm; (C) CCK‐8 assay for evaluating chondrocyte proliferation in control and AGEs groups. Experiments were repeated three times. *Indicates comparison between groups; **p < 0.01, ***p < 0.001, ****p < 0.0001. [file ACEL-25-e70515-s007.jpg]

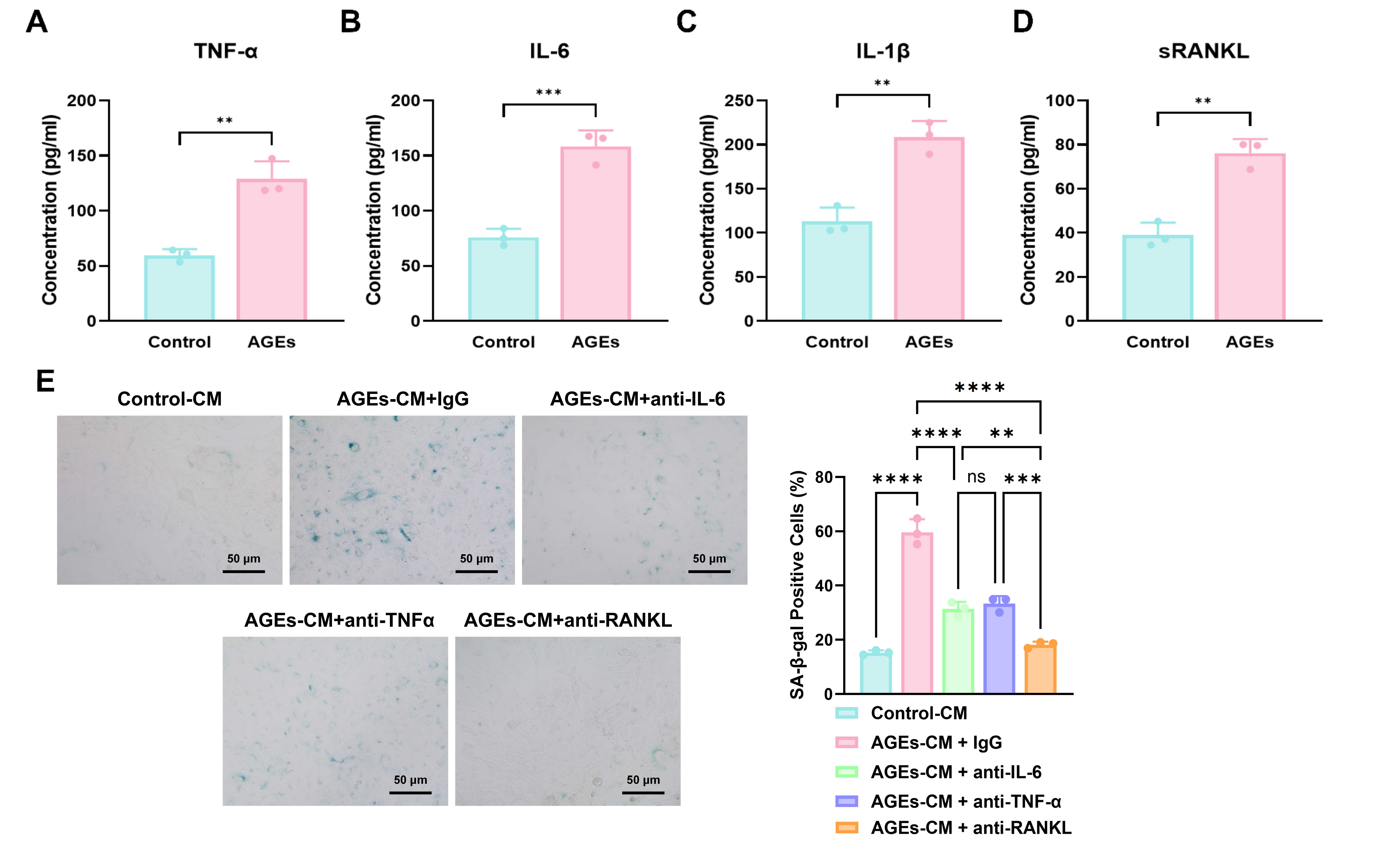

Supplement: Supplementary file 6 — Figure S6: Analysis of osteoclast‐derived paracrine mediators and their contribution to chondrocyte senescence. (A–D) ELISA quantification of IL‐6, TNF‐α, IL‐1β, and soluble RANKL (sRANKL) levels in the conditioned medium of osteoclasts (OC‐CM) from Control and AGEs groups. (E) Representative SA‐β‐gal staining images of chondrocytes treated with AGEs‐OC‐CM. Scale bar: 50 μm. Data are presented as mean ± SD from three independent experiments. *p < 0.05, **p < 0.01 versus Control‐CM group; #p < 0.05, ##p < 0.01 versus AGEs‐CM + IgG group. [file ACEL-25-e70515-s006.jpg]

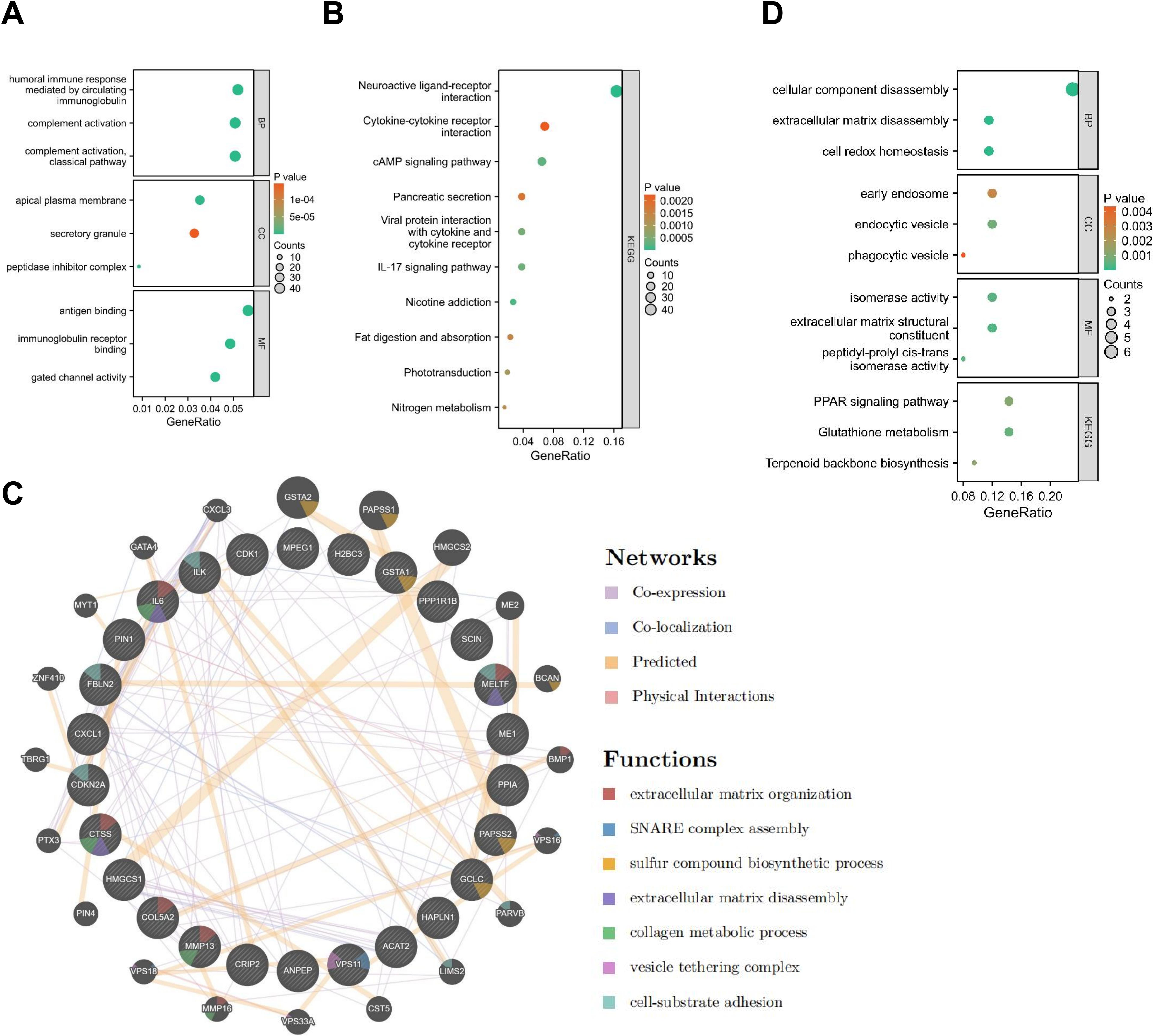

Supplement: Supplementary file 7 — Figure S7: Extended enrichment and network analyses of transcriptomic and proteomic alterations. (A) GO enrichment analysis of RNA‐seq data; (B) KEGG pathway enrichment analysis of differentially expressed genes; (C) PPI network of differentially expressed proteins; (D) GO and KEGG enrichment analyses of differentially expressed proteins. [file ACEL-25-e70515-s001.jpg]

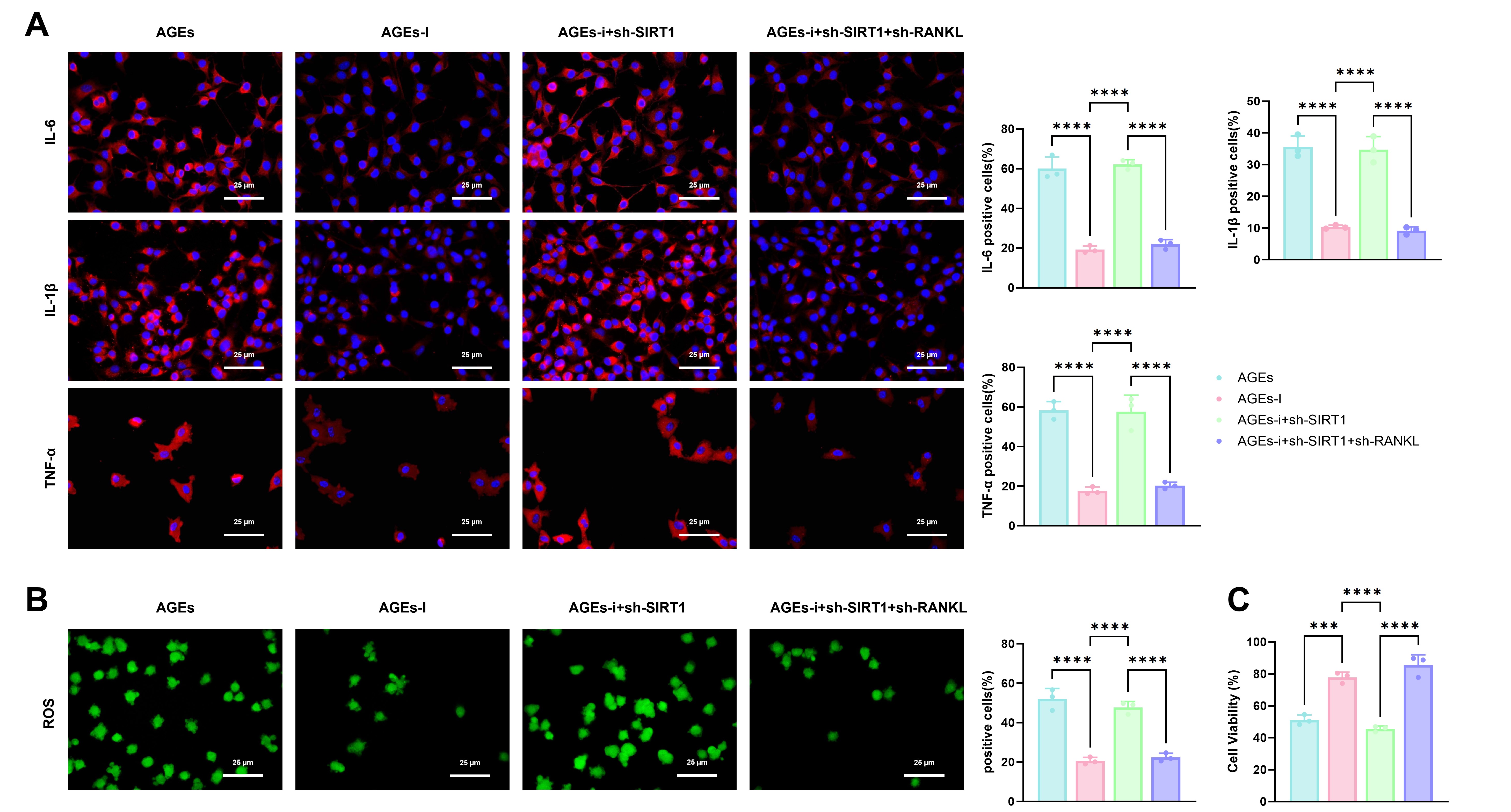

Supplement: Supplementary file 8 — Figure S8: Effects of AGEs on chondrocyte SASP and function via the SIRT1/RANKL/RANK pathway. (A) IF analysis of SASP markers TNF‐α, IL‐6, and IL‐1β in chondrocytes from AGEs, AGEs‐i, AGEs‐i+sh‐SIRT1, and AGEs‐i+sh‐SIRT1+sh‐RANKL groups, bar = 25 μm; (B) ROS assay detecting ROS activity in chondrocytes from the same groups, bar = 25 μm; (C) CCK‐8 assay assessing chondrocyte proliferation in the same groups. Experiments were repeated three times. *Indicates comparison between groups; **p < 0.01, ***p < 0.001, ****p < 0.0001. [file ACEL-25-e70515-s010.jpg]

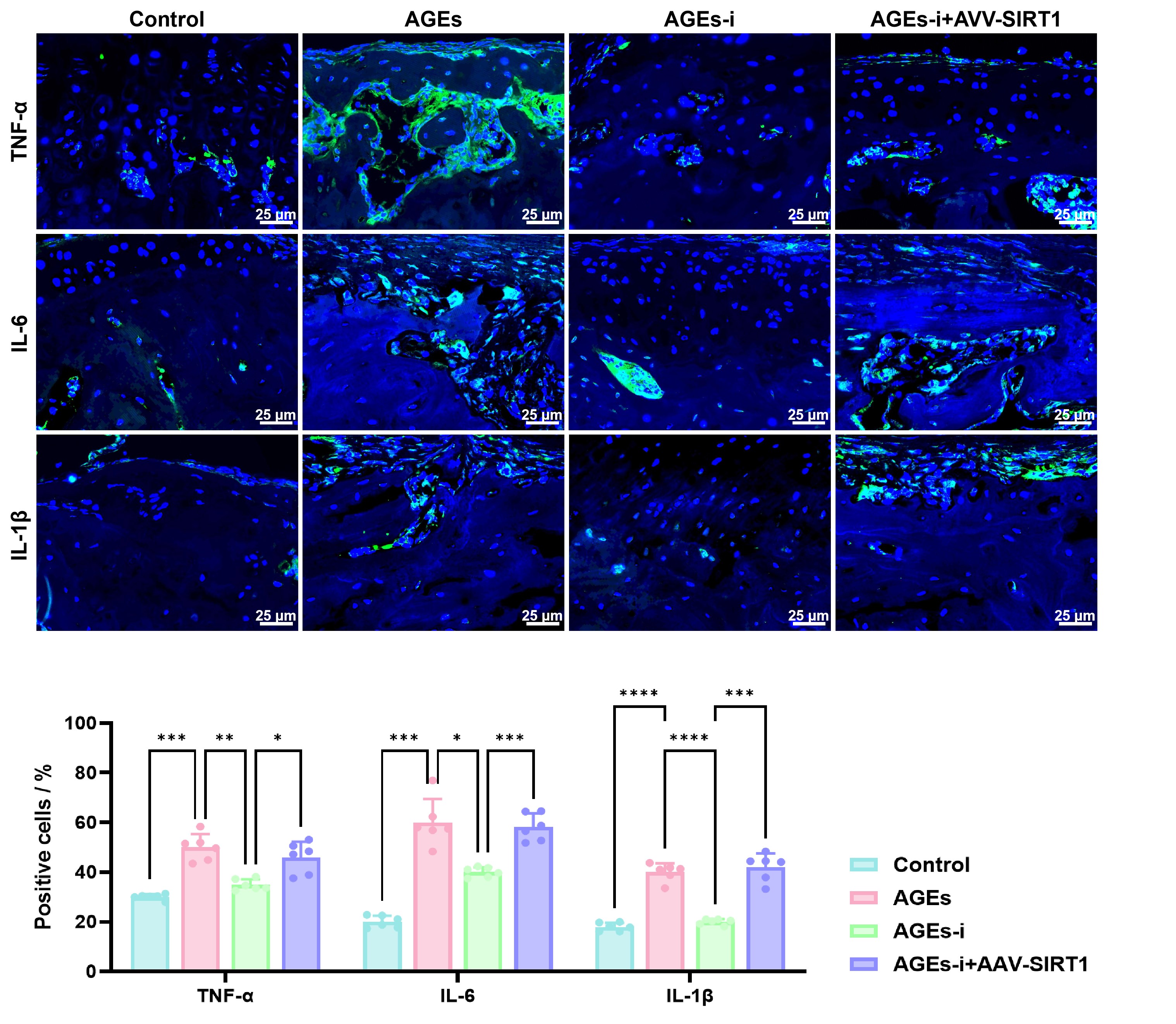

Supplement: Supplementary file 9 — Figure S9: In vivo validation of AGEs/SIRT1/RANKL/RANK signaling in regulating chondrocyte SASP. IF analysis of SASP markers TNF‐α, IL‐6, and IL‐1β in chondrocytes from control, AGEs, AGEs‐i, and AGEs‐i+AAV‐SIRT1 groups, bar = 25 μm. Each group included six mice. *Indicates comparison between groups; **p < 0.01, ***p < 0.001, ****p < 0.0001. [file ACEL-25-e70515-s003.jpg]
