# Supplementary material for: SIRT1 Downregulation by Advanced Glycation End Products Activates RANKL‐Dependent Osteoclast Signaling and Drives Chondrocyte Senescence During Osteoarthritis Development
Source: Aging Cell. 2026 May 1;25(5):e70515. doi: 10.1111/acel.70515 (PMC13134948; doi:10.1111/acel.70515)
Supplement: Supplementary file 10 — Table S1: Sequence of RT‐qPCR. Table S2: Primary antibody information. Table S3: Sequence of shRNA. [file ACEL-25-e70515-s005.docx]

**Table S1. Sequence of RT-qPCR.**

| **Name** | **Sequence (5’-3’)** |
| --- | --- |
| SIRT1(mouse) | F: AGCGGCTTGAGGGTAATCAA |
|  | R: TCCAGATCCTCCAGCACATT |
| RANKL(mouse) | F: TACTTTCGAGCGCAGATGGAT |
|  | R: GGGCCACATCCAACCATGAG |
| RANK(mouse) | F: TTCCTCTAAGTGCACTCCTACCT |
|  | R: ATCCTTGTTGAGAGGGATGC |
| GAPDH (mouse) | F: CCCTTAAGAGGGATGCTGCC |
|  | R: ACTGTGCCGTTGAATTTGCC |

Note: Abbreviations: F, forward; R, reverse.

**Table S2. Primary antibody information.**

| **Name** | **Manufactor** | **Cat.NO** | **Dilution ratio** |
| --- | --- | --- | --- |
| SIRT1 (Rabbit) | Cell Signaling Technology | 2028S | 1:1000 |
| RANKL (Rabbit) | Proteintech | 66610-1-Ig | 1:2000 |
| RANK | Abcam | ab305233 | 1: 1000 |
| NF-κB (Rabbit) | Cell Signaling Technology | 8242T | 1:1000 |
| p- NF-κB (Rabbit) | Cell Signaling Technology | 3033T | 1:1000 |
| JNK (Rabbit) | Cell Signaling Technology | 9252T | 1:1000 |
| p-JNK (Rabbit) | Cell Signaling Technology | 4668T | 1:1000 |
| p38 MAPK (Rabbit) | Cell Signaling Technology | 8690T | 1:1000 |
| p-p38 MAPK (Rabbit) | Cell Signaling Technology | 9211S | 1:1000 |
| GAPDH | Abcam | ab181602 | 1:10000 |

**Table S3. Sequence of shRNA.**

| **Name** | **Sequence (5’-3’)** |
| --- | --- |
| sh-NC | GTTTATTACCTATACCTTAATC |
| sh-SIRT1-1 | GCAAAGCCTTTCTGAATCTAT |
| sh-SIRT1-2 | CCTCGAACAATTCTTAAAGAT |
| sh-RANKL-2 | CGCAGATGGATCCTAACAGAA |
| sh- RANKL -2 | CCCAAGTTCTCATAACCTGAT |
